# Supplementary material for: Prediction of excess pregnancy weight gain using psychological, physical, and social predictors: A validated model in a prospective cohort study
Source: PLoS One. 2020 Jun 2;15(6):e0233774. doi: 10.1371/journal.pone.0233774 (PMC7266315; doi:10.1371/journal.pone.0233774)
Supplement: S1 File — (DOCX) [file pone.0233774.s001.docx]

**S1 File. Previously explored factors and novel factors in pregnancy for gestational weight gain**

| 1. Construct | **Previously explored factors in our systematic review** | **Previously *un*explored factors in pregnancy** |
| --- | --- | --- |
| Cognition | *Weight locus of control*, *target weight gain*, *weight attitudes*, *body image*, *Barriers to Healthy Eating*, self-efficacy | *Nutrition knowledge*, *normative factors* |
| Affect | No association between general affect & pregnancy weight gain | *Pregnancy-related anxiety* |
| Personality | Not adequately studied | *Impulse control*, *perfectionism*, *motivation*, *expressive suppression* & *Big 5 Factors* |
| Behaviour | *Dietary restraint;* Other behaviours: *diet*, *physical activity*, *sleep*, *smoking*, *eating in front of a screen* | *Binge Eating Scale*, *night eating, emotional eating* |
