# Supplementary material for: Prediction of excess pregnancy weight gain using psychological, physical, and social predictors: A validated model in a prospective cohort study
Source: PLoS One. 2020 Jun 2;15(6):e0233774. doi: 10.1371/journal.pone.0233774 (PMC7266315; doi:10.1371/journal.pone.0233774)
Supplement: S2 File — (PDF) [file pone.0233774.s002.pdf]

McMaster University

Thank you for sharing your thoughts on pregnancy and weight gain.

We invite you to share your thoughts on pregnancy and weight gain. Your **participation is very important and greatly appreciated.** The information gathered from this study will hopefully be able to help improve health care and the health of women and children.

This study, which will take only about **15 minutes to complete,** is being conducted by researchers at McMaster University.

Your participation is completely voluntary, and you are free to withdraw at any time. Please complete the survey and return it to the staff.

Thank you for your participation.

Today's date: __ __ __ (month) __ __ (day) 201 ___ (year)

PART A

Date your baby is due: __ __ __ (month) __ __ (day) 201 ___ (year)

1. During this pregnancy, who has provided most of your pregnancy care?

   (*Please fill in **ONE** circle only.)*

   ❍ Obstetrician
   ❍ Family physician
   ❍ Midwife
   ❍ Nurse practitioner
   ❍ Other (*Please specify*) ____

2. What is your age? ___ ___ years

3. Is this your first time giving birth?

   ❍ No → How many times have you already given birth? _____ (times)
   ❍ Yes

4. What is your height? ____ feet ____ inches **OR** ____ cm

5. How much weight do you plan to gain in total this pregnancy? _____ pounds **OR** _____ kg

6. What was your weight right before this pregnancy? _____ pounds **OR** _____ kg

7. How *satisfied* were you with your weight before this pregnancy? *(Please circle **ONE** number that best reflects your feelings.)*

Not satisfied at all	Not very satisfied	Somewhat satisfied	Very satisfied
1	2	3	4

8. How would you classify your weight just <u>before</u> this pregnancy?
*(Please circle **ONE** number that best reflects your feelings.)*

**Markedly underweight**	**Underweight**	**Normal weight**	**Overweight**	**Markedly Overweight**
1	2	3	4	5

9. Has your doctor, midwife or nurse made a recommendation about how much weight you should gain **in total** during pregnancy?

❍ NO → how much do you think you should gain in total? _______ pounds **OR** ________ kg
❍ YES → how much? _______ pounds **OR** ________ kg **OR** ❍ I can't remember

10. How much **weight gain** do you think is **recommended** for you during the 1st trimester (which finishes at the end of the 13th week in pregnancy)? (*Your best guess is fine.*) ____ pounds **OR** ____ kg

11. Do you believe that there are any risks to gaining **too little weight** during pregnancy?

a. for you? ❍ No
❍ Yes

b. for the baby? ❍ No
❍ Yes

12. Do you believe that there are any risks to gaining **too much weight** during pregnancy?

a. for you? ❍ No
❍ Yes

b. for the baby? ❍ No
❍ Yes

13. This section deals with **your** attitudes toward weight gain during pregnancy.
*(Please circle **ONE** number on each row of the table below that best reflects your feelings.)*

	Strongly disagree	Disagree	Neither disagree nor agree	Agree	Strongly agree
Whether my weight changes is up to me.	1	2	3	4	5
If I eat right, and can get enough exercise and rest, I can control my weight the way I want.	1	2	3	4	5
Being the right weight is mainly good luck.	1	2	3	4	5
You can't control the amount of weight you gain when you are pregnant.	1	2	3	4	5

14. *Please circle **ONE** number on each row of the table below that best reflects your thoughts on* to what extent your **<u>family and friends</u> agree or disagree with the following statements**.

**Women who are pregnant . . .**	Strongly Disagree	Disagree	Neither Agree nor Disagree	Agree	Strongly Agree
. . .need to eat two times as much as before pregnancy.	1	2	3	4	5
. . .crave foods more intensely than other people.	1	2	3	4	5
. . .should eat what they crave.	1	2	3	4	5
. . .should not exert themselves physically.	1	2	3	4	5
. . .should not be worried about gaining too much weight during pregnancy.	1	2	3	4	5

15. Do you have any chronic health problems (physical or psychiatric problems for at least 6 months that have been diagnosed by a health professional)?

❍ I have no chronic health problems

OR

(*Please fill in circles for **ALL** that apply.)*

❍ Depression
❍ Anxiety
❍ "Low" or "underactive" thyroid
❍ "High" or "overactive" thyroid
❍ Diabetes before pregnancy
❍ Other(s) (*Please list all*) ________________________________

16. Before this pregnancy, did you smoke?

*(Please fill in **ONE** circle only.)*

❍ No
❍ Yes

17. Do you smoke now?

*(Please fill in **ONE** circle only.)*

❍ No
❍ Yes

18. What is your highest level of education?

(*Please fill in **ONE** circle only.)*

❍ Some high school or less
❍ Completed high school
❍ Community college or technical school (some or completed)
❍ Undergraduate university (some or completed)
❍ Graduate degree

19. What is your best estimate of the total income, before taxes and deductions, of all people in your household, from all sources in the past 12 months?

(*Please fill in **ONE** circle only.)*

❍ Under $10,000
❍ $10,000 – 19,999
❍ $20,000 – 39,999
❍ $40,000 – 59,999
❍ $60,000 – 79,999
❍ Over $80,000
❍ I prefer not to answer

20. What is your marital status?

(*Please fill in **ONE** circle only.)*

❍ Married
❍ Common-Law
❍ Living with a partner
❍ Single (never married)
❍ Divorced
❍ Widowed

21. Which of the following best describes your racial background?

(*Please fill in **ONE** circle only.)*

❍ Aboriginal (e.g. Inuit, Métis, First Nations, etc)
❍ Arab (e.g. Armenian, Egyptian, Iranian, Lebanese, Moroccan, etc)
❍ Black (e.g. African, Haitian, Jamaican, Somali, etc)
❍ Chinese
❍ Filipino
❍ Japanese
❍ Korean
❍ Latin American
❍ South Asian (e.g. East Indian, Pakistani, Punjabi, Sri Lankan, etc)
❍ South East Asian (e.g. Cambodian, Indonesian, etc)
❍ White (Caucasian)
❍ Other (*Please specify*) ___________________________________

PART B

Please answer the following questions about your typical routine during this pregnancy.

1. Do you eat meals in front of a screen (such as watching television, or using a computer, PDA, iPad or gaming device)?
   - ❍ No
   - ❍ Yes → (*Please fill in **ONE** square only.)*
     - ☐ every meal
     - ☐ most meals
     - ☐ some meals
     - ☐ almost *no* meals
2. Do you have a television set in your bedroom?
   - ❍ No
   - ❍ Yes → Do you watch television before going to sleep? (*Please fill in **ONE** square only.)*
     - ☐ every night
     - ☐ most nights
     - ☐ some nights
     - ☐ never
3. During a typical day, do you drink soda pop, cola or juice?
   - ❍ No
   - ❍ Yes → how many glasses or cans on average each day? ____
4. On average, how many times would you eat fast food such as pizza, Taco Bell, Dairy Queen, McDonald's, Burger King, Wendy's etc.?
   - ❍ 1 time per month (or less)
   - ❍ 2 or 3 times per month
   - ❍ 1 time per week
   - ❍ 2 or 3 times per week
   - ❍ Most days
5. On average, how many times would you eat snack foods such as candy, cookies, chips, donuts, etc?
   - ❍ 1 time per month (or less)
   - ❍ 2 or 3 times per month
   - ❍ 1 time per week
   - ❍ 2 or 3 times per week
   - ❍ 1 time per day
   - ❍ 2 times per day
   - ❍ 3 times per day
6. On average, how many servings of fruits and vegetable do you eat per day (not counting juice, French fries or other fried foods)?

   ___ ___
7. How much of your **daily food intake** do you eat ***after*** suppertime (including during the middle of the night)?
   - ❍ less than a quarter (less than ¼)
   - ❍ at least a quarter (¼ or more)

8. When you get **up out of bed in the middle of the night**, *on average* how often do you snack?

❍ Never
❍ 1 time per week
❍ 2 or more times per week

**During this pregnancy. . .**	Never	Rarely	Often	Always
Do you have feelings of guilt after overeating?	0	1	2	3
Do you ever feel that when you started eating you just couldn't stop?	0	1	2	3

PART C

Listed below are some statements concerning **food**.

*(Please circle **ONE** number on each line of the table below that best reflects your feel*

	Strongly Disagree	Disagree	Don't know	Agree	Strongly Agree	Not applicable as I am vegan or vegetarian
1. I don't buy milk because it costs too much.	1	2	3	4	5	0
2. I don't buy fruits and vegetables because they cost too much.	1	2	3	4	5	

PART D

Listed below are some statements concerning your feelings towards **food and exercise**.

*(Please circle **ONE** number on each line of the table below that best reflects your feelings.)*

**How sure or unsure are you that during pregnancy you can**:	Very unsure	Unsure	Neither unsure nor sure	Sure	Very sure
1. Eat foods that are good for you even when family or social life takes a lot of your time.	1	2	3	4	5
2. Get regular exercise.	1	2	3	4	5

PART E

Listed below are a number of statements concerning **personal attitudes and characteristics**. *There are no right or wrong answers and no trick questions. Work quickly and do not think too long about the exact meaning of the question.*

*(Please circle **ONE** number on each row of the table below that best reflects your feelings.)*

	Almost Never	Sometimes	About half the time	Most of the time	Almost Always
1. I control my emotions by not expressing them.	1	2	3	4	5
2. When I'm upset, I have difficulty controlling my behaviour.	1	2	3	4	5
3. When I'm upset, it takes me a long time to feel better.	1	2	3	4	5
**Part E continued...**	Almost Never	Sometimes	About half the time	Most of the time	Almost Always
4. When I'm upset, I believe that there's nothing I can do to make myself feel better.	1	2	3	4	5
5. When I'm upset, I know that I can find a way to eventually feel better.	1	2	3	4	5
6. When I'm upset, I become embarrassed for feeling that way.	1	2	3	4	5
7. I feel that I must do things perfectly or not do them at all.	1	2	3	4	5

PART F

The following questions deal with your **attitudes toward weight gain and dieting *in the 3 months before* learning you were pregnant**.

*(Please circle **ONE** number on each line of the table below that best reflects your feelings.)*

	Never	Rarely	Often	Always
1. How often were you dieting?	0	1	2	3
2. How often did you limit your carbohydrate and sugar intake?	0	1	2	3
3. Did you have feelings of guilt after overeating?	0	1	2	3
4. Did you ever feel that when you started eating you just couldn't stop?	0	1	2	3
5. Do you want to eat when you are emotionally upset?	0	1	2	3

6. Before you were pregnant, would a weight change of 5 lb (2.3 kg) affect the way you live your life?

Not at all	Slightly	Moderately	Very much
0	1	2	3

7. During the 3 months before pregnancy did you use any of the following strategies to maintain or lose weight?

Exercising to the point that it interfered with other activities	❍ NO<br>❍ YES
Vomiting, diet pills, laxatives, diuretics, syrup of ipecac, enemas	❍ NO<br>❍ YES

PART G

The following questions are concerned with your **eating behaviours**.

*(Please circle **ONE** number on each line of the table below that best reflects your feelings.)*

	Never	Sometimes	Regularly	Often	Always
1. I eat sensibly when with others, but overdo so when I'm alone.	0	1	2	3	4
2. If others saw how much I ate, then I'd feel ashamed.	0	1	2	3	4

**When I am considering eating more than I should or when I am considering eating a food that I feel is not particularly healthy, I tell myself it is okay because:**	Never	Rarely	Frequently	Always
3. I will eat healthier later.	0	1	2	3
4. I will compensate by eating less later.	0	1	2	3
5. I will compensate by doing some exercise later.	0	1	2	3

6. I have a lot of fear regarding the health of my baby. ❍ No
❍ Yes → If yes, how do you cope with your fears:
- ☐ Eating more
- ☐ Eating less
- ☐ Other ________

PART H

Read each of the following statements and select the appropriate number to indicate how it best describes you. Please be as honest and accurate as possible in your responses. There are no right or wrong answers.

	Very unlike me	Somewhat unlike me	Neither like me nor unlike me	Somewhat like me	Very much like me
1. I'm just not the goal-setting type.	1	2	3	4	5
2. I like to set goals and work toward them.	1	2	3	4	5

PART I

These questions are part of a scale commonly used to **assess depression. Like all your answers, they will be kept completely confidential, but please speak with your care provider if you have concerns.**

*(Please circle **ONE** number on each line of the table below that best reflects your feelings.)*

In the <u>2 weeks before your current pregnancy</u>, how often were you bothered by any of the following problems?	Not at all	Several days	More than half the days	Nearly every day
1. Not being able to stop or control worrying	0	1	2	3
2. Feeling down, depressed, or hopeless	0	1	2	3

PART J

The following questions deal with **pregnancy symptoms during this pregnancy.**

	Never	About once a week	About once a day	Several times a day	Many times a day
1. At its worst, please describe your nausea related to pregnancy.	0	1	2	3	4

If you responded "Never" please go to ***Question 2****.*

a. How do (did) you usually cope with the nausea?

(*Fill in circles for* ***ALL*** *that apply.)*

❍ Eat something
❍ Avoid eating
❍ Distract myself
❍ Smoke
❍ Other(s) (*Please list all*) ________________________________

	Never	About once a week	About once a day	Several times a day	Many times a day
2. Do you have food cravings related to pregnancy?	0	1	2	3	4

If you responded "Never", please go to ***Part K****.*

a. What types of food do you crave regularly?

(*Fill in circles for* ***ALL*** *that apply.)*

❍ Sweets
❍ Salty snacks (e.g. chips, nuts, etc.)
❍ Meat (e.g. chicken, fish, steak, etc.)
❍ Carbohydrates (e.g. bread, pasta, rice, etc.)
❍ Fruits
❍ Vegetables
❍ Other(s) (*Please list all*) ________________________________

b. How do you usually cope with the cravings?

(*Fill in circles for* ***ALL*** *that apply.)*

❍ Eat what I crave
❍ Avoid what I crave/ Ignore the cravings
❍ Distract myself
❍ Smoke
❍ Other(s) (*Please list all*) ________________________________

PART K

Please *estimate about* how much time you spend on groups of activities on an **average weekday**. If you normally sleep 7½ hours, please mark the 30-minute box and 7-hour box.

	Examples		Hours	Minutes
A	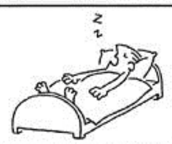	Sleep, rest	☐1 ☐2 ☐3 ☐4 ☐5 ☐6 ☐7 ☐8 ☐9 ☐10	☐15 ☐30 ☐45
B	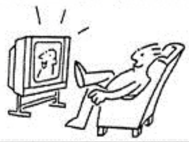	Sitting watching TV, reading	☐1 ☐2 ☐3 ☐4 ☐5 ☐6 ☐7 ☐8 ☐9 ☐10	☐15 ☐30 ☐45
C	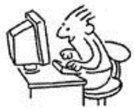	Working at a computer, sitting in a meeting, eating	☐1 ☐2 ☐3 ☐4 ☐5 ☐6 ☐7 ☐8 ☐9 ☐10	☐15 ☐30 ☐45
D	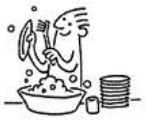	Standing, washing dishes, driving	☐1 ☐2 ☐3 ☐4 ☐5 ☐6 ☐7 ☐8 ☐9 ☐10	☐15 ☐30 ☐45
E	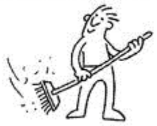	Light cleaning, shopping with grocery cart, walking downstairs	☐1 ☐2 ☐3 ☐4 ☐5 ☐6 ☐7 ☐8 ☐9 ☐10	☐15 ☐30 ☐45
F	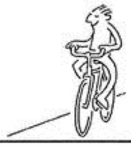	Brisk walking, bicycling	☐1 ☐2 ☐3 ☐4 ☐5 ☐6 ☐7 ☐8 ☐9 ☐10	☐15 ☐30 ☐45
G	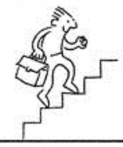	Gardening, carrying objects upstairs	☐1 ☐2 ☐3 ☐4 ☐5 ☐6 ☐7 ☐8 ☐9 ☐10	☐15 ☐30 ☐45
H	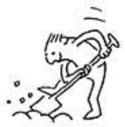	Aerobics, health club exercise	☐1 ☐2 ☐3 ☐4 ☐5 ☐6 ☐7 ☐8 ☐9 ☐10	☐15 ☐30 ☐45
I	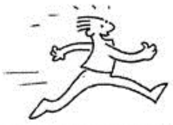	More effort than level H: running, racing on a bicycle	☐1 ☐2 ☐3 ☐4 ☐5 ☐6 ☐7 ☐8 ☐9 ☐10	☐15 ☐30 ☐45

PART L

Below are a number of **personality traits that may or may not apply to yourself**. *Using the following scale, Please indicate the extent to which you agree or disagree with each statement. You should rate the extent to which the pair of traits applies to yourself, even if one characteristic applies more strongly than the other.*
(Please circle ***ONE*** *number on each line of the table below that best reflects your feelings.)*

**I see myself as:**	Disagree strongly	Disagree moderately	Disagree a little	Neither agree nor disagree	Agree a little	Agree moderately	Agree strongly
1. Extraverted, enthusiastic	1	2	3	4	5	6	7
2. Critical, quarrelsome	1	2	3	4	5	6	7
3. Dependable, self-disciplined	1	2	3	4	5	6	7
4. Anxious, easily upset	1	2	3	4	5	6	7
5. Open to new experiences, complex	1	2	3	4	5	6	7
6. Reserved, quiet	1	2	3	4	5	6	7
7. Sympathetic, warm	1	2	3	4	5	6	7
8. Disorganized, careless	1	2	3	4	5	6	7
9. Calm, emotionally stable	1	2	3	4	5	6	7
10. Conventional, uncreative	1	2	3	4	5	6	7

PART M

Please circle the silhouette that most accurately shows your body size as you perceived it before pregnancy. Please be honest.

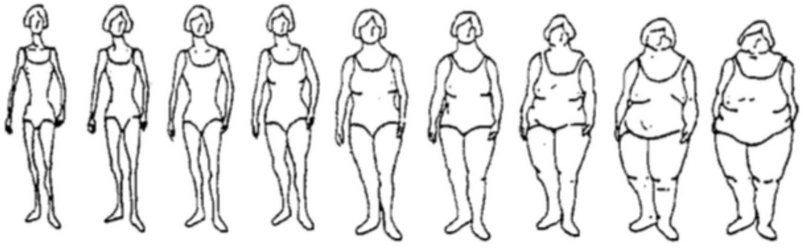

Please circle the silhouette that most accurately depicts the body size that you would have preferred before pregnancy. Again, please be honest.

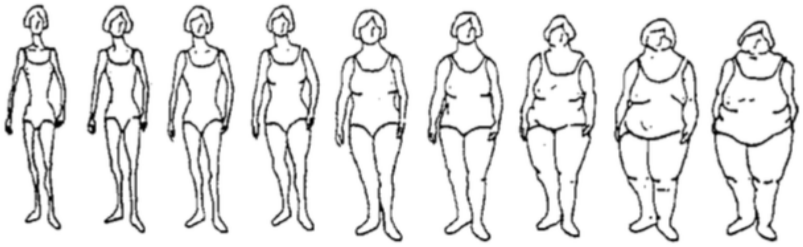

PART N

If there is anything else you would like to tell us concerning the survey, please write it here:

____

If any of the topics in this survey have raised concerns or questions, please discuss them with your care provider.

THANK YOU FOR YOUR PARTICIPATION!

If you would be interested in answering a similar survey at about 32 weeks of your pregnancy, would you please provide us your email so we can send a link to the survey then? All surveys completed at that point will receive a ToysRUs or Chapters (your choice) $10 gift card.

☐☐☐☐☐☐☐☐☐☐☐☐☐ @ ☐☐☐☐☐☐☐ ☐ (your email)
