# Supplementary material for: Prediction of excess pregnancy weight gain using psychological, physical, and social predictors: A validated model in a prospective cohort study
Source: PLoS One. 2020 Jun 2;15(6):e0233774. doi: 10.1371/journal.pone.0233774 (PMC7266315; doi:10.1371/journal.pone.0233774)
Supplement: S3 File — BMI = body mass index; n = number; SD = standard deviation; wk = weeks; yr = years. (DOCX) [file pone.0233774.s003.docx]

| **Characteristics Mean (SD) / n (%)** | **Missing (n=338)** | | **Non-missing (n=632)** | | ***p* values** |
| --- | --- | --- | --- | --- | --- |
| **Maternal age, yr** | 30.3 | (5.1) | 30.6 | (4.8) | 0.430 |
| **Gestational age at survey, wk** | 15.0 | (3.5) | 14.7 | (3.4) | 0.143 |
| **Prepregnancy BMI** |  |  |  |  | 0.214 |
| Underweight (BMI <18.5 kg/m^2^) | 15 | (4.4) | 14 | (2.2) |  |
| Normal weight (BMI 18.5-24.9 kg/m^2^) | 165 | (48.8) | 328 | (51.9) |  |
| Overweight (BMI 25.0-29.9 kg/m^2^) | 85 | (25.1) | 147 | (23.3) |  |
| Obese (BMI ≥30 kg/m^2^) | 73 | (21.6) | 143 | (22.6) |  |
| **Pregnancy weight gain** |  |  |  |  | 0.984 |
| Below guidelines | 54 | (16.0) | 100 | (15.8) |  |
| Within guidelines | 96 | (28.4) | 183 | (29.0) |  |
| Above guidelines | 188 | (55.6) | 349 | (55.2) |  |

**S3 File. Comparison of major characteristics between women with and without missing data in prospective cohort** **pregnancy weight gain study**

BMI = body mass index; n = number; SD = standard deviation; wk = weeks; yr = years
