# Supplementary material for: Prediction of excess pregnancy weight gain using psychological, physical, and social predictors: A validated model in a prospective cohort study
Source: PLoS One. 2020 Jun 2;15(6):e0233774. doi: 10.1371/journal.pone.0233774 (PMC7266315; doi:10.1371/journal.pone.0233774)
Supplement: S4 File — Data are means (standard deviation) and number of participants (percentage). Percentages may not total 100 due to rounding.BMI, body mass index; TPB, theory of planned behavior. (DOCX) [file pone.0233774.s004.docx]

**S4 File.** **Differences in exposure variables among study participants in prospective cohort pregnancy weight gain study by pregnancy weight gain status**

| **Exposure variables** (n = 970) | | **Excess gestational weight gain** | | | | | | ***p* value** |
| --- | --- | --- | --- | --- | --- | --- | --- | --- |
|  |  | **No** | | | **Yes** | | |  |
|  |  | n | (n=433) | | n | (n=537) | |  |
| **Maternal age, year, mean (SD)** | | 433 | 30.9 | (4.8) | 537 | 30.2 | (4.9) | 0.031 |
| **Race** | |  |  |  |  |  |  | 0.010 |
|  | White |  | 309 | (71.4%) |  | 427 | (79.5%) |  |
|  | Non-white |  | 123 | (28.4) |  | 108 | (20.1) |  |
|  | Not reported |  | 1 | (0.2) |  | 2 | (0.4) |  |
| **Marital status** | |  |  |  |  |  |  | 0.444 |
|  | Married, common-law, or living with a partner |  | 402 | (92.8) |  | 496 | (92.4) |  |
|  | Single, divorced, or widowed |  | 31 | (7.2) |  | 39 | (7.3) |  |
|  | Not reported |  | 0 | (0.0) |  | 2 | (0.4) |  |
| **Education** | |  |  |  |  |  |  | 0.615 |
|  | Community college or technical school or lower |  | 181 | (41.8) |  | 231 | (43.0) |  |
|  | Undergraduate university or higher |  | 252 | (58.2) |  | 305 | (56.8) |  |
|  | Not reported |  | 0 | (0.0) |  | 1 | (0.2) |  |
| **Household income** | |  |  |  |  |  |  | 0.099 |
|  | < $40,000 |  | 73 | (16.9) |  | 79 | (14.7) |  |
|  | $40,000 - $79,999 |  | 103 | (23.8) |  | 147 | (27.4) |  |
|  | ≥ $80,000 |  | 224 | (51.7) |  | 251 | (46.7) |  |
|  | Not reported |  | 33 | (7.6) |  | 60 | (11.2) |  |
| **Smoking** | |  |  |  |  |  |  | 0.006 |
|  | Never |  | 363 | (83.8) |  | 414 | (77.1) |  |
|  | Before this pregnancy |  | 39 | (9.0) |  | 85 | (15.8) |  |
|  | During this pregnancy |  | 29 | (6.7) |  | 38 | (7.1) |  |
|  | Not reported |  | 2 | (0.5) |  | 0 | (0.0) |  |
| **Parity** | |  |  |  |  |  |  | 0.105 |
|  | 0 |  | 210 | (48.5) |  | 296 | (55.1) |  |
|  | 1+ |  | 220 | (50.8) |  | 239 | (44.5) |  |
|  | Not reported |  | 3 | (0.7) |  | 2 | (0.4) |  |
| **Prepregnancy BMI** | |  |  |  |  |  |  | <0.001 |
|  | Underweight (BMI <18.5 kg/m^2^) |  | 21 | (4.8) |  | 8 | (1.5) |  |
|  | Normal weight (BMI 18.5-24.9 kg/m^2^) |  | 275 | (63.5) |  | 218 | (40.6) |  |
|  | Overweight (BMI 25.0-29.9 kg/m^2^) |  | 56 | (12.9) |  | 176 | (32.8) |  |
|  | Obese (BMI ≥30 kg/m^2^) |  | 81 | (18.7) |  | 135 | (25.1) |  |
| **Depression** | |  |  |  |  |  |  | 0.070 |
|  | No |  | 411 | (94.9) |  | 494 | (92.0) |  |
|  | **Yes** |  | 22 | (5.1) |  | 43 | (8.0) |  |
| **Anxiety** | |  |  |  |  |  |  | 0.492 |
|  | No |  | 389 | (89.8) |  | 475 | (88.5) |  |
|  | Yes |  | 44 | (10.2) |  | 62 | (11.5) |  |
| **Other chronic health conditions** | |  |  |  |  |  |  | 0.839 |
|  | No |  | 317 | (73.2) |  | 390 | (72.6) |  |
|  | Yes |  | 116 | (26.8) |  | 147 | (27.4) |  |
| **Satisfied with weight before pregnancy** | |  |  |  |  |  |  | 0.001 |
|  | Not very satisfied or not satisfied at all |  | 125 | (28.9) |  | 215 | (40.0) |  |
|  | Somewhat satisfied or very satisfied |  | 303 | (70.0) |  | 319 | (59.4) |  |
|  | Not reported |  | 5 | (1.2) |  | 3 | (0.6) |  |
| **Planned total gestational weight gain** | |  |  |  |  |  |  | <0.001 |
|  | Not reported |  | 31 | (7.2) |  | 34 | (6.3) |  |
|  | Within guidelines |  | 165 | (38.1) |  | 171 | (31.8) |  |
|  | Below guidelines |  | 159 | (36.7) |  | 110 | (20.5) |  |
|  | Above guidelines |  | 78 | (18.0) |  | 222 | (41.3) |  |
| **Weight gain recommendation levels by healthcare provider** | |  |  |  |  |  |  | 0.202 |
|  | None |  | 338 | (78.1) |  | 424 | (79.0) |  |
|  | Within guidelines |  | 44 | (10.2) |  | 38 | (7.1) |  |
|  | Below guidelines |  | 16 | (3.7) |  | 15 | (2.8) |  |
|  | Above guidelines |  | 12 | (2.8) |  | 24 | (4.5) |  |
|  | Not reported/I can’t remember |  | 23 | (5.3) |  | 36 | (6.7) |  |
| **Perceived weight gain recommendation for the 1^st^ trimester** | |  |  |  |  |  |  | 0.332 |
|  | None |  | 14 | (3.2) |  | 17 | (3.2) |  |
|  | Within guidelines |  | 133 | (30.7) |  | 142 | (26.4) |  |
|  | Outside guidelines |  | 286 | (66.1) |  | 378 | (70.4) |  |
| **Do you believe that there any risks for you to gaining too little weight during pregnancy?** | |  |  |  |  |  |  | 0.237 |
|  | Yes |  | 174 | (40.2) |  | 231 | (43.0) |  |
|  | No |  | 258 | (59.6) |  | 301 | (56.1) |  |
|  | Not reported |  | 1 | (0.2) |  | 5 | (0.9) |  |
| **Do you believe that there are any risks for the baby to gaining too little weight during pregnancy?** | |  |  |  |  |  |  | 0.553 |
|  | Yes |  | 84 | (19.4) |  | 113 | (21.0) |  |
|  | No |  | 347 | (80.1) |  | 419 | (78.0) |  |
|  | Not reported |  | 2 | (0.5) |  | 5 | (0.9) |  |
| **Do you believe that there any risks for you to gaining too much weight during pregnancy?** | |  |  |  |  |  |  | 0.679 |
|  | Yes |  | 27 | (6.2) |  | 35 | (6.5) |  |
|  | No |  | 404 | (93.3) |  | 497 | (92.6) |  |
|  | Not reported |  | 2 | (0.5) |  | 5 | (0.9) |  |
| **Do you believe that there any risks for the baby to gaining too much weight during pregnancy?** | |  |  |  |  |  |  | 0.920 |
|  | Yes |  | 46 | (10.6) |  | 57 | (10.6) |  |
|  | No |  | 384 | (88.7) |  | 475 | (88.5) |  |
|  | Not reported |  | 3 | (0.7) |  | 5 | (0.9) |  |
| **Whether my weight changes is up to me** | |  |  |  |  |  |  | 0.643 |
|  | Disagree or strongly disagree |  | 138 | (31.9) |  | 190 | (35.4) |  |
|  | Neither disagree nor agree |  | 118 | (27.3) |  | 138 | (25.7) |  |
|  | Agree or strongly agree |  | 172 | (39.7) |  | 205 | (38.2) |  |
|  | Not reported |  | 5 | (1.2) |  | 4 | (0.7) |  |
| **If I eat right, and can get enough exercise and rest, I can control my weight the way I want** | |  |  |  |  |  |  | 0.599 |
|  | Disagree or strongly disagree |  | 71 | (16.4) |  | 99 | (18.4) |  |
|  | Neither disagree nor agree |  | 78 | (18.0) |  | 106 | (19.7) |  |
|  | Agree or strongly agree |  | 281 | (64.9) |  | 330 | (61.5) |  |
|  | Not reported |  | 3 | (0.7) |  | 2 | (0.4) |  |
| **Being the right weight is mainly good luck** | |  |  |  |  |  |  | 0.625 |
|  | Agree or strongly agree |  | 34 | (7.9) |  | 41 | (7.6) |  |
|  | Neither disagree nor agree |  | 81 | (18.7) |  | 106 | (19.7) |  |
|  | Disagree or strongly disagree |  | 311 | (71.8) |  | 386 | (71.9) |  |
|  | Not reported |  | 7 | (1.6) |  | 4 | (0.7) |  |
| **You can’t control the amount of weight you gain when you are pregnant** | |  |  |  |  |  |  | 0.163 |
|  | Agree or strongly agree |  | 51 | (11.8) |  | 86 | (16.0) |  |
|  | Neither disagree nor agree |  | 87 | (20.1) |  | 118 | (22.0) |  |
|  | Disagree or strongly disagree |  | 291 | (67.2) |  | 330 | (61.5) |  |
|  | Not reported |  | 4 | (0.9) |  | 3 | (0.6) |  |
| **Think that family and friends believe that pregnant women need to eat two times as much as before pregnancy** | |  |  |  |  |  |  | 0.112 |
|  | Disagree or strongly disagree |  | 327 | (75.5) |  | 417 | (77.7) |  |
|  | Neither disagree nor agree |  | 51 | (11.8) |  | 43 | (8.0) |  |
|  | Agree or strongly agree |  | 52 | (12.0) |  | 76 | (14.2) |  |
|  | Not reported |  | 3 | (0.7) |  | 1 | (0.2) |  |
| **Think that family and friends believe that pregnant women crave foods more intensely than other people** | |  |  |  |  |  |  | 0.370 |
|  | Disagree or strongly disagree |  | 56 | (12.9) |  | 55 | (10.2) |  |
|  | Neither disagree nor agree |  | 65 | (15.0) |  | 96 | (17.9) |  |
|  | Agree or strongly agree |  | 308 | (71.1) |  | 383 | (71.3) |  |
|  | Not reported |  | 4 | (0.9) |  | 3 | (0.6) |  |
| **Think that family and friends believe that pregnant women should eat what they crave** | |  |  |  |  |  |  | 0.428 |
|  | Disagree or strongly disagree |  | 136 | (31.4) |  | 177 | (33.0) |  |
|  | Neither disagree nor agree |  | 163 | (37.6) |  | 197 | (36.7) |  |
|  | Agree or strongly agree |  | 130 | (30.0) |  | 162 | (30.2) |  |
|  | Not reported |  | 4 | (0.9) |  | 1 | (0.2) |  |
| **Think that family and friends believe that pregnant women should not exert themselves physically** | |  |  |  |  |  |  | 0.089 |
|  | Disagree or strongly disagree |  | 146 | (33.7) |  | 203 | (37.8) |  |
|  | Neither disagree nor agree |  | 72 | (16.6) |  | 110 | (20.5) |  |
|  | Agree or strongly agree |  | 210 | (48.5) |  | 220 | (41.0) |  |
|  | Not reported |  | 5 | (1.2) |  | 4 | (0.7) |  |
| **Think that family and friends believe that pregnant women should not be worried about gaining too much weight during pregnancy** | |  |  |  |  |  |  | 0.708 |
|  | Disagree or strongly disagree |  | 221 | (51.0) |  | 265 | (49.3) |  |
|  | Neither disagree nor agree |  | 83 | (19.2) |  | 95 | (17.7) |  |
|  | Agree or strongly agree |  | 125 | (28.9) |  | 173 | (32.2) |  |
|  | Not reported |  | 4 | (0.9) |  | 4 | (0.7) |  |
| **How often do you eat meals in front of a screen?** | |  |  |  |  |  |  | <0.001 |
|  | None or almost no meals |  | 167 | (38.6) |  | 141 | (26.3) |  |
|  | Some meals |  | 188 | (43.4) |  | 295 | (54.9) |  |
|  | Most meals or more |  | 72 | (16.6) |  | 93 | (17.3) |  |
|  | Not reported |  | 6 | (1.4) |  | 8 | (1.5) |  |
| **How often do you watch television before going to sleep?** | |  |  |  |  |  |  | 0.347 |
|  | None |  | 274 | (63.3) |  | 318 | (59.2) |  |
|  | Some nights |  | 71 | (16.4) |  | 105 | (19.6) |  |
|  | Most nights or more |  | 85 | (19.6) |  | 106 | (19.7) |  |
|  | Not reported |  | 3 | (0.7) |  | 8 | (1.5) |  |
| **During a typical day, do you drink soda pop, cola, or juice?** | |  |  |  |  |  |  | 0.769 |
|  | No |  | 254 | (58.7) |  | 303 | (56.4) |  |
|  | Yes |  | 178 | (41.1) |  | 233 | (43.4) |  |
|  | Not reported |  | 1 | (0.2) |  | 1 | (0.2) |  |
| **On average, how many times would you eat fast food?** | |  |  |  |  |  |  | 0.023 |
|  | ≤1 time/month |  | 154 | (35.6) |  | 149 | (27.7) |  |
|  | 2-3 times/month |  | 145 | (33.5) |  | 202 | (37.6) |  |
|  | ≥1 time/week |  | 132 | (30.5) |  | 186 | (34.6) |  |
|  | Not reported |  | 2 | (0.5) |  | 0 | (0.0) |  |
| **Fruit and vegetable intake per day** | |  |  |  |  |  |  | 0.748 |
|  | < 5 servings/day |  | 238 | (55.0) |  | 291 | (54.2) |  |
|  | ≥ 5 servings/day |  | 187 | (43.2) |  | 239 | (44.5) |  |
|  | Not reported |  | 8 | (1.8) |  | 7 | (1.3) |  |
| **On average, how many times would you eat snack foods?** | |  |  |  |  |  |  | 0.089 |
|  | ≤1 time/week |  | 193 | (44.6) |  | 223 | (41.5) |  |
|  | ≥2 times/week |  | 237 | (54.7) |  | 314 | (58.5) |  |
|  | Not reported |  | 3 | (0.7) |  | 0 | (0.0) |  |
| **How much of your daily food intake do you eat after suppertime?** | |  |  |  |  |  |  | 0.082 |
|  | <¼ |  | 399 | (92.1) |  | 501 | (93.3) |  |
|  | ≥¼ |  | 30 | (6.9) |  | 36 | (6.7) |  |
|  | Not reported |  | 4 | (0.9) |  | 0 | (0.0) |  |
| **Do you snack in the middle of the night?** | |  |  |  |  |  |  | 0.747 |
|  | No |  | 377 | (87.1) |  | 465 | (86.6) |  |
|  | Yes |  | 52 | (12.0) |  | 69 | (12.8) |  |
|  | Not reported |  | 4 | (0.9) |  | 3 | (0.6) |  |
| **During this pregnancy, do you have feelings of guilt after overeating?** | |  |  |  |  |  |  | 0.018 |
|  | Never or rarely |  | 347 | (80.1) |  | 430 | (80.1) |  |
|  | Often or always |  | 65 | (15.0) |  | 97 | (18.1) |  |
|  | Not reported |  | 21 | (4.8) |  | 10 | (1.9) |  |
| **During this pregnancy, do you ever feel that when you started eating you just couldn’t stop?** | |  |  |  |  |  |  | 0.049 |
|  | Never or rarely |  | 383 | (88.5) |  | 470 | (87.5) |  |
|  | Often or always |  | 32 | (7.4) |  | 56 | (10.4) |  |
|  | Not reported |  | 18 | (4.2) |  | 11 | (2.0) |  |
| **During pregnancy, you can eat foods that are good for you even when family or social life takes a lot of your time** | |  |  |  |  |  |  | 0.471 |
|  | Unsure or very unsure |  | 22 | (5.1) |  | 38 | (7.1) |  |
|  | Neither unsure or sure |  | 42 | (9.7) |  | 58 | (10.8) |  |
|  | Sure or very sure |  | 366 | (84.5) |  | 439 | (81.8) |  |
|  | Not reported |  | 3 | (0.7) |  | 2 | (0.4) |  |
| **During pregnancy, you can get regular exercise** | |  |  |  |  |  |  | 0.515 |
|  | Unsure or very unsure |  | 52 | (12.0) |  | 63 | (11.7) |  |
|  | Neither unsure or sure |  | 50 | (11.5) |  | 78 | (14.5) |  |
|  | Sure or very sure |  | 328 | (75.8) |  | 394 | (73.4) |  |
|  | Not reported |  | 3 | (0.7) |  | 2 | (0.4) |  |
| **I control my emotions by not expressing them** | |  |  |  |  |  |  | 0.428 |
|  | Almost never or sometimes |  | 325 | (75.1) |  | 415 | (77.3) |  |
|  | About half the time |  | 74 | (17.1) |  | 78 | (14.5) |  |
|  | Most of the time or almost always |  | 34 | (7.9) |  | 42 | (7.8) |  |
|  | Not reported |  | 0 | (0.0) |  | 2 | (0.4) |  |
| **When I am upset, I have difficulty controlling my behaviour** | |  |  |  |  |  |  | 0.990 |
|  | Almost never or sometimes |  | 361 | (83.4) |  | 450 | (83.8) |  |
|  | About half the time |  | 40 | (9.2) |  | 47 | (8.8) |  |
|  | Most of the time or almost always |  | 27 | (6.2) |  | 33 | (6.1) |  |
|  | Not reported |  | 5 | (1.2) |  | 7 | (1.3) |  |
| **When I’m upset, it takes me a long time to feel better** | |  |  |  |  |  |  | 0.198 |
|  | Almost never or sometimes |  | 340 | (78.5) |  | 421 | (78.4) |  |
|  | About half the time |  | 58 | (13.4) |  | 65 | (12.1) |  |
|  | Most of the time or almost always |  | 27 | (6.2) |  | 47 | (8.8) |  |
|  | Not reported |  | 8 | (1.8) |  | 4 | (0.7) |  |
| **When I’m upset, I believe that there’s nothing I can do to make myself feel better** | |  |  |  |  |  |  | 0.948 |
|  | Almost never or sometimes |  | 399 | (92.1) |  | 497 | (92.6) |  |
|  | About half the time |  | 25 | (5.8) |  | 30 | (5.6) |  |
|  | Most of the time or almost always |  | 8 | (1.8) |  | 8 | (1.5) |  |
|  | Not reported |  | 1 | (0.2) |  | 2 | (0.4) |  |
| **When I'm upset, I know I can find a way to eventually feel better** | |  |  |  |  |  |  | 0.839 |
|  | Most of the time or almost always |  | 357 | (82.4) |  | 431 | (80.3) |  |
|  | About half the time |  | 35 | (8.1) |  | 51 | (9.5) |  |
|  | Almost never or sometimes |  | 39 | (9.0) |  | 52 | (9.7) |  |
|  | Not reported |  | 2 | (0.5) |  | 3 | (0.6) |  |
| **When I am upset, I become embarrassed for feeling that way** | |  |  |  |  |  |  | 0.987 |
|  | Almost never or sometimes |  | 352 | (81.3) |  | 433 | (80.6) |  |
|  | About half the time |  | 45 | (10.4) |  | 59 | (11.0) |  |
|  | Most of the time or almost always |  | 34 | (7.9) |  | 42 | (7.8) |  |
|  | Not reported |  | 2 | (0.5) |  | 3 | (0.6) |  |
| **I feel that I must do things perfectly or not do them at all** | |  |  |  |  |  |  | 0.712 |
|  | Almost never or sometimes |  | 326 | (75.3) |  | 389 | (72.4) |  |
|  | About half the time |  | 50 | (11.5) |  | 74 | (13.8) |  |
|  | Most of the time or almost always |  | 54 | (12.5) |  | 71 | (13.2) |  |
|  | Not reported |  | 3 | (0.7) |  | 3 | (0.6) |  |
| **During the 3 months before pregnancy, how often were you dieting?** | |  |  |  |  |  |  | 0.003 |
|  | Never or rarely |  | 367 | (84.8) |  | 410 | (76.4) |  |
|  | Often or always |  | 66 | (15.2) |  | 125 | (23.3) |  |
|  | Not reported |  | 0 | (0.0) |  | 2 | (0.4) |  |
| **During the 3 months before pregnancy, how often did you limit your carbohydrate and sugar intake?** | |  |  |  |  |  |  | 0.053 |
|  | Never or rarely |  | 271 | (62.6) |  | 297 | (55.3) |  |
|  | Often or always |  | 162 | (37.4) |  | 239 | (44.5) |  |
|  | Not reported |  | 0 | (0.0) |  | 1 | (0.2) |  |
| **During the 3 months before pregnancy, did you have feelings of guilt after overeating?** | |  |  |  |  |  |  | 0.019 |
|  | Never or rarely |  | 312 | (72.1) |  | 343 | (63.9) |  |
|  | Often or always |  | 121 | (27.9) |  | 193 | (35.9) |  |
|  | Not reported |  | 0 | (0.0) |  | 1 | (0.2) |  |
| **During the 3 months before pregnancy, did you ever feel that when you started eating you just couldn’t stop?** | |  |  |  |  |  |  | 0.298 |
|  | Never or rarely |  | 393 | (90.8) |  | 471 | (87.7) |  |
|  | Often or always |  | 39 | (9.0) |  | 65 | (12.1) |  |
|  | Not reported |  | 1 | (0.2) |  | 1 | (0.2) |  |
| **During the 3 months before pregnancy, did you want to eat when you were emotionally upset?** | |  |  |  |  |  |  | 0.002 |
|  | Never or rarely |  | 333 | (76.9) |  | 358 | (66.7) |  |
|  | Often or always |  | 99 | (22.9) |  | 178 | (33.1) |  |
|  | Not reported |  | 1 | (0.2) |  | 1 | (0.2) |  |
| **I eat sensibly when with others, but overdo so when I’m alone** | |  |  |  |  |  |  | 0.001 |
|  | No |  | 268 | (61.9) |  | 270 | (50.3) |  |
|  | Yes |  | 163 | (37.6) |  | 264 | (49.2) |  |
|  | Not reported |  | 2 | (0.5) |  | 3 | (0.6) |  |
| **If others saw how much I ate, then I’d feel ashamed** | |  |  |  |  |  |  | 0.011 |
|  | No |  | 355 | (82.0) |  | 398 | (74.1) |  |
|  | Yes |  | 75 | (17.3) |  | 136 | (25.3) |  |
|  | Not reported |  | 3 | (0.7) |  | 3 | (0.6) |  |
| **When I am considering eating more than I should or when I am considering eating a food that I feel is not particularly healthy, I tell myself it is okay because I will eat healthier later.** | |  |  |  |  |  |  | 0.045 |
|  | Never |  | 113 | (26.1) |  | 105 | (19.6) |  |
|  | Rarely, frequently, or always |  | 317 | (73.2) |  | 426 | (79.3) |  |
|  | Not reported |  | 3 | (0.7) |  | 6 | (1.1) |  |
| **When I am considering eating more than I should or when I am considering eating a food that I feel is not particularly healthy, I tell myself it is okay because I will compensate by eating less later.** | |  |  |  |  |  |  | 0.665 |
|  | Never |  | 183 | (42.3) |  | 214 | (39.9) |  |
|  | Rarely, frequently, or always |  | 246 | (56.8) |  | 316 | (58.8) |  |
|  | Not reported |  | 4 | (0.9) |  | 7 | (1.3) |  |
| **When I am considering eating more than I should or when I am considering eating a food that I feel is not particularly healthy, I tell myself it is okay because I will compensate by doing some exercise later.** | |  |  |  |  |  |  | 0.102 |
|  | Never |  | 123 | (28.4) |  | 121 | (22.5) |  |
|  | Rarely, frequently, or always |  | 306 | (70.7) |  | 412 | (76.7) |  |
|  | Not reported |  | 4 | (0.9) |  | 4 | (0.7) |  |
| **I have a lot of fear regarding the health of my baby** | |  |  |  |  |  |  | 0.168 |
|  | No |  | 328 | (75.8) |  | 386 | (71.9) |  |
|  | Yes |  | 94 | (21.7) |  | 142 | (26.4) |  |
|  | Not reported |  | 11 | (2.5) |  | 9 | (1.7) |  |
| **Nausea related to pregnancy** | |  |  |  |  |  |  | 0.280 |
|  | Never or 1 time/week |  | 112 | (25.9) |  | 133 | (24.8) |  |
|  | ≥ 1 time/day |  | 321 | (74.1) |  | 401 | (74.7) |  |
|  | Not reported |  | 0 | (0.0) |  | 3 | (0.6) |  |
| **Food cravings related to pregnancy** | |  |  |  |  |  |  | 0.137 |
|  | Never or 1 time/week |  | 276 | (63.7) |  | 338 | (62.9) |  |
|  | ≥ 1 time/day |  | 145 | (33.5) |  | 193 | (35.9) |  |
|  | Not reported |  | 12 | (2.8) |  | 6 | (1.1) |  |
| **Eat something to cope with nausea** | |  |  |  |  |  |  | 0.455 |
|  | No |  | 196 | (45.3) |  | 256 | (47.7) |  |
|  | Yes |  | 237 | (54.7) |  | 281 | (52.3) |  |
| **Avoiding eating to cope with nausea** | |  |  |  |  |  |  | 0.152 |
|  | No |  | 338 | (78.1) |  | 439 | (81.8) |  |
|  | Yes |  | 95 | (21.9) |  | 98 | (18.2) |  |
| **Take medication to cope with nausea** | |  |  |  |  |  |  | 0.205 |
|  | None |  | 81 | (18.7) |  | 114 | (21.2) |  |
|  | Medication use |  | 33 | (7.6) |  | 54 | (10.1) |  |
|  | Other ways |  | 319 | (73.7) |  | 369 | (68.7) |  |
| **Eat what I crave to cope with cravings** | |  |  |  |  |  |  | 0.816 |
|  | No |  | 132 | (30.5) |  | 160 | (29.8) |  |
|  | Yes |  | 301 | (69.5) |  | 377 | (70.2) |  |
| **Avoid what I crave to cope with cravings** | |  |  |  |  |  |  | 0.916 |
|  | No |  | 378 | (87.3) |  | 470 | (87.5) |  |
|  | Yes |  | 55 | (12.7) |  | 67 | (12.5) |  |
| **Distract myself to cope with cravings** | |  |  |  |  |  |  | 0.583 |
|  | No |  | 352 | (81.3) |  | 429 | (79.9) |  |
|  | Yes |  | 81 | (18.7) |  | 108 | (20.1) |  |
| **Sleep** | |  |  |  |  |  |  | 0.592 |
|  | < 8 hr/day |  | 189 | (43.6) |  | 222 | (41.3) |  |
|  | ≥ 8 hr/day |  | 239 | (55.2) |  | 311 | (57.9) |  |
| **Total physical activity** | |  |  |  |  |  |  | 0.575 |
|  | Low |  | 139 | (32.1) |  | 158 | (29.4) |  |
|  | Medium |  | 126 | (29.1) |  | 173 | (32.2) |  |
|  | High |  | 137 | (31.6) |  | 161 | (30.0) |  |
|  | Not reported |  | 31 | (7.2) |  | 45 | (8.4) |  |
| **Preferred body size image before pregnancy** | |  |  |  |  |  |  | 0.022 |
|  | Underweight |  | 93 | (21.5) |  | 75 | (14.0) |  |
|  | Normal weight |  | 307 | (70.9) |  | 421 | (78.4) |  |
|  | Overweight or obese |  | 31 | (7.2) |  | 38 | (7.1) |  |
|  | Not reported |  | 2 | (0.5) |  | 3 | (0.6) |  |
| **Comparison between perceived and preferred body size image** | |  |  |  |  |  |  | 0.017 |
|  | Actually smaller than preferred |  | 26 | (6.0) |  | 20 | (3.7) |  |
|  | Accurate |  | 151 | (34.9) |  | 148 | (27.6) |  |
|  | Actually larger than preferred |  | 254 | (58.7) |  | 366 | (68.2) |  |
|  | Not reported |  | 2 | (0.5) |  | 3 | (0.6) |  |
| **Comparison between BMI and perceived body size image** | |  |  |  |  |  |  | <0.001 |
|  | Actually smaller than perceived |  | 19 | (4.4) |  | 13 | (2.4) |  |
|  | Accurate |  | 273 | (63.0) |  | 270 | (50.3) |  |
|  | Actually larger than perceived |  | 140 | (32.3) |  | 251 | (46.7) |  |
|  | Not reported |  | 1 | (0.2) |  | 3 | (0.6) |  |
| **Sitting time/day, hr** | | 402 | 6.5 | (3.1) | 492 | 6.6 | (3.1) | 0.465 |
| **TPB score** | | 430 | 3.8 | (1.0) | 533 | 3.8 | (1.0) | 0.809 |
| **Personality- Extraversion** | | 423 | 4.4 | (1.5) | 529 | 4.4 | (1.5) | 0.732 |
| **Personality- Agreeableness** | | 419 | 5.3 | (1.0) | 526 | 5.4 | (1.0) | 0.052 |
| **Personality- Conscientiousness** | | 421 | 5.8 | (1.0) | 530 | 5.6 | (1.1) | 0.017 |
| **Personality- Emotional Stability** | | 423 | 4.8 | (1.3) | 530 | 4.8 | (1.3) | 0.537 |
| **Personality- Openness** | | 424 | 5.1 | (1.1) | 532 | 5.1 | (1.1) | 0.945 |

Data are means (standard deviation) and number of participants (percentage). Percentages may not total 100 due to rounding.

BMI, body mass index; TPB, theory of planned behavior.
