# Supplementary material for: Prediction of excess pregnancy weight gain using psychological, physical, and social predictors: A validated model in a prospective cohort study
Source: PLoS One. 2020 Jun 2;15(6):e0233774. doi: 10.1371/journal.pone.0233774 (PMC7266315; doi:10.1371/journal.pone.0233774)
Supplement: S5 File — BMI, body mass index; HCP, health care provider; TPB, theory of planned behavior. (DOCX) [file pone.0233774.s005.docx]

**S5 File. Univariable logistic regression analysis of predictors of excess pregnancy weight gain in a prospective cohort study**

| **Predictors** (n = 970) | | **Complete case analysis**  **OR (95% CI)** |
| --- | --- | --- |
| **Maternal age, yr** | |  |
|  | n | 970 |
|  | *p* value | 0.031 |
|  | Continuous variable | 0.97 (0.95, 1.00) |
| **Race** | |  |
|  | n | 967 |
|  | *p* value | 0.003 |
|  | White | 1.0 (reference) |
|  | Non-white | 0.64 (0.47, 0.86) |
| **Marital status** | |  |
|  | n | 968 |
|  | *p* value | 0.938 |
|  | Married, common-law, or living with a partner | 1.0 (reference) |
|  | Single, divorced, or widowed | 1.02 (0.62, 1.66) |
| **Education** | |  |
|  | n | 969 |
|  | *p* value | 0.685 |
|  | Community college or technical school or lower | 1.0 (reference) |
|  | Undergraduate university or higher | 0.95 (0.73, 1.23) |
| **Household income** | |  |
|  | n | 877 |
|  | *p* value | 0.250 |
|  | < $40,000 | 1.0 (reference) |
|  | $40,000 - $79,999 | 1.32 (0.88, 1.98) |
|  | > $80,000 | 1.04 (0.72, 1.49) |
| **Smoking** | |  |
|  | n | 968 |
|  | *p* value | 0.007 |
|  | None | 1.0 (reference) |
|  | Before this pregnancy | 1.91 (1.28, 2.86) |
|  | During this pregnancy | 1.15 (0.69, 1.90) |
| **Parity** | |  |
|  | n | 965 |
|  | *p* value | 0.045 |
|  | 0 | 1.0 (reference) |
|  | 1+ | 0.77 (0.60, 0.99) |
| **Prepregnancy BMI** | |  |
|  | n | 970 |
|  | *p* value | <0.0001 |
|  | Underweight (BMI <18.5 kg/m^2^) | 0.48 (0.21, 1.11) |
|  | Normal weight (BMI 18.5-24.9 kg/m^2^) | 1.0 (reference) |
|  | Overweight (BMI 25.0-29.9 kg/m^2^) | 3.96 (2.80, 5.62) |
|  | Obese (BMI ≥30 kg/m^2^) | 2.10 (1.51, 2.92) |
| **Depression** | |  |
|  | n | 970 |
|  | *p* value | 0.072 |
|  | No | 1.0 (reference) |
|  | Yes | 1.63 (0.96, 2.76) |
| **Anxiety** | |  |
|  | n | 970 |
|  | *p* value | 0.492 |
|  | No | 1.0 (reference) |
|  | Yes | 1.15 (0.77, 1.74) |
| **Other chronic health conditions** | |  |
|  | n | 970 |
|  | *p* value | 0.839 |
|  | No | 1.0 (reference) |
|  | Yes | 1.03 (0.77, 1.37) |
| **Preterm birth** | |  |
|  | n | 966 |
|  | *p* value | 0.786 |
|  | No | 1.0 (reference) |
|  | Yes | 0.93 (0.53, 1.61) |
| **Satisfied with weight before pregnancy** | |  |
|  | n | 962 |
|  | *p* value | <0.0001 |
|  | Not satisfied at all or not very satisfied | 1.0 (reference) |
|  | Somewhat satisfied or very satisfied | 0.61 (0.47, 0.80) |
| **Planned total gestational weight gain** | |  |
|  | n | 970 |
|  | *p* value | <0.0001 |
|  | None | 1.06 (0.62, 1.80) |
|  | Within guidelines | 1.0 (reference) |
|  | Below guidelines | 0.67 (0.48, 0.92) |
|  | Above guidelines | 2.75 (1.96, 3.84) |
| **Weight gain recommendation levels by healthcare provider** | |  |
|  | n | 970 |
|  | *p* value | 0.207 |
|  | None | 1.45 (0.92, 2.29) |
|  | Within guidelines | 1.0 (reference) |
|  | Below guidelines | 1.09 (0.48, 2.48) |
|  | Above guidelines | 2.32 (1.02, 5.25) |
|  | Not reported/I can’t remember | 1.82 (0.92, 3.58) |
| **Perceived weight gain recommendation for the 1^st^ trimester** | |  |
|  | n | 970 |
|  | *p* value | 0.332 |
|  | None | 1.14 (0.54, 2.40) |
|  | Within guidelines | 1.0 (reference) |
|  | Outside guidelines | 1.24 (0.93, 1.64) |
| **Do you believe that there any risks for you to gaining too little weight during pregnancy?** | |  |
|  | n | 964 |
|  | *p* value | 0.326 |
|  | No | 1.0 (reference) |
|  | Yes | 0.88 (0.68, 1.14) |
| **Do you believe that there are any risks for the baby to gaining too little weight during pregnancy?** | |  |
|  | n | 963 |
|  | *p* value | 0.503 |
|  | No | 1.0 (reference) |
|  | Yes | 0.90 (0.65, 1.23) |
| **Do you believe that there any risks for you to gaining too much weight during pregnancy?** | |  |
|  | n | 963 |
|  | *p* value | 0.844 |
|  | No | 1.0 (reference) |
|  | Yes | 0.95 (0.56, 1.59) |
| **Do you believe that there any risks for the baby to gaining too much weight during pregnancy?** | |  |
|  | n | 962 |
|  | *p* value | 0.993 |
|  | No | 1.0 (reference) |
|  | Yes | 1.00 (0.66, 1.51) |
| **Whether my weight changes is up to me** | |  |
|  | n | 961 |
|  | *p* value | 0.539 |
|  | Disagree or strongly disagree | 1.18 (0.85, 1.64) |
|  | Neither disagree nor agree | 1.0 (reference) |
|  | Agree or strongly agree | 1.02 (0.74, 1.40) |
| **If I eat right, and can get enough exercise and rest, I can control my weight the way I want** | |  |
|  | n | 965 |
|  | *p* value | 0.498 |
|  | Disagree or strongly disagree | 1.03 (0.67, 1.57) |
|  | Neither disagree nor agree | 1.0 (reference) |
|  | Agree or strongly agree | 0.86 (0.62, 1.21) |
| **Being the right weight is mainly good luck** | |  |
|  | n | 959 |
|  | *p* value | 0.938 |
|  | Agree or strongly agree | 0.92 (0.54, 1.58) |
|  | Neither disagree nor agree | 1.0 (reference) |
|  | Disagree or strongly disagree | 0.95 (0.68, 1.31) |
| **You can’t control the amount of weight you gain when you are pregnant** | |  |
|  | n | 963 |
|  | *p* value | 0.097 |
|  | Agree or strongly agree | 1.24 (0.80, 1.94) |
|  | Neither disagree nor agree | 1.0 (reference) |
|  | Disagree or strongly disagree | 0.84 (0.61, 1.15) |
| **Think that family and friends believe that pregnant women need to eat two times as much as before pregnancy** | |  |
|  | n | 966 |
|  | *p* value | 0.108 |
|  | Disagree or strongly disagree | 1.51 (0.98, 2.33) |
|  | Neither disagree nor agree | 1.0 (reference) |
|  | Agree or strongly agree | 1.73 (1.01, 2.97) |
| **Think that family and friends believe that pregnant women crave foods more intensely than other people** | |  |
|  | n | 963 |
|  | *p* value | 0.260 |
|  | Disagree or strongly disagree | 0.66 (0.41, 1.08) |
|  | Neither disagree nor agree | 1.0 (reference) |
|  | Agree or strongly agree | 0.84 (0.59, 1.19) |
| **Think that family and friends believe that pregnant women should eat what they crave** | |  |
|  | n | 965 |
|  | *p* value | 0.893 |
|  | Disagree or strongly disagree | 1.08 (0.79, 1.46) |
|  | Neither disagree nor agree | 1.0 (reference) |
|  | Agree or strongly agree | 1.03 (0.76, 1.41) |
| **Think that family and friends believe that pregnant women should not exert themselves physically** | |  |
|  | n | 961 |
|  | *p* value | 0.048 |
|  | Disagree or strongly disagree | 0.91 (0.63, 1.31) |
|  | Neither disagree nor agree | 1.0 (reference) |
|  | Agree or strongly agree | 0.69 (0.48, 0.98) |
| **Think that family and friends believe that pregnant women should not be worried about gaining too much weight during pregnancy** | |  |
|  | n | 962 |
|  | *p* value | 0.523 |
|  | Disagree or strongly disagree | 1.05 (0.74, 1.48) |
|  | Neither disagree nor agree | 1.0 (reference) |
|  | Agree or strongly agree | 1.21 (0.83, 1.76) |
| **Do you eat meals in front of a screen?** | |  |
|  | n | 967 |
|  | *p* value | <0.0001 |
|  | No | 1.0 (reference) |
|  | Yes | 1.74 (1.29, 2.34) |
| **How often do you eat meals in front of a screen?** | |  |
|  | n | 956 |
|  | *p* value | <0.0001 |
|  | None or almost no meals | 1.0 (reference) |
|  | Some meals | 1.86 (1.39, 2.48) |
|  | Most meals or more | 1.53 (1.05, 2.24) |
| **Do you have a television set in your bedroom?** | |  |
|  | n | 968 |
|  | *p* value | 0.125 |
|  | No | 1.0 (reference) |
|  | Yes | 1.22 (0.95, 1.58) |
| **How often do you watch television before going to sleep?** | |  |
|  | n | 959 |
|  | *p* value | 0.378 |
|  | None | 1.0 (reference) |
|  | Some nights | 1.27 (0.91, 1.79) |
|  | Most nights or more | 1.07 (0.77, 1.49) |
| **During a typical day, do you drink soda pop, cola, or juice?** | |  |
|  | n | 968 |
|  | *p* value | 0.479 |
|  | No | 1.0 (reference) |
|  | Yes | 1.10 (0.85, 1.42) |
| **On average, how many times would you eat fast food?** | |  |
|  | n | 968 |
|  | *p* value | 0.029 |
|  | ≤1 time/month | 1.0 (reference) |
|  | 2-3 times/month | 1.44 (1.06, 1.96) |
|  | ≥1 time/week | 1.46 (1.06, 2.00) |
| **Fruit and vegetable intake per day** | |  |
|  | n | 955 |
|  | *p* value | 0.735 |
|  | < 5 servings/day | 1.0 (reference) |
|  | ≥ 5 servings/day | 1.05 (0.81, 1.35) |
| **On average, how many times would you eat snack foods?** | |  |
|  | n | 967 |
|  | *p* value | 0.295 |
|  | ≤1 time/week | 1.0 (reference) |
|  | ≥2 times/week | 1.15 (0.89, 1.48) |
| **How much of your daily food intake do you eat after suppertime?** | |  |
|  | n | 966 |
|  | *p* value | 0.859 |
|  | <¼ | 1.0 (reference) |
|  | ≥¼ | 0.96 (0.58, 1.58) |
| **Do you snack in the middle of the night?** | |  |
|  | n | 963 |
|  | *p* value | 0.711 |
|  | No | 1.0 (reference) |
|  | Yes | 1.08 (0.73, 1.58) |
| **During this pregnancy, do you have feelings of guilt after overeating?** | |  |
|  | n | 939 |
|  | *p* value | 0.290 |
|  | Never or rarely | 1.0 (reference) |
|  | Often or always | 1.20 (0.85, 1.70) |
| **During this pregnancy, do you ever feel that when you started eating you just couldn’t stop?** | |  |
|  | n | 941 |
|  | *p* value | 0.126 |
|  | Never or rarely | 1.0 (reference) |
|  | Often or always | 1.43 (0.90, 2.25) |
| **During pregnancy, you can eat foods that are good for you even when family or social life takes a lot of your time** | |  |
|  | n | 965 |
|  | *p* value | 0.362 |
|  | Unsure or very unsure | 1.25 (0.65, 2.42) |
|  | Neither unsure or sure | 1.0 (reference) |
|  | Sure or very sure | 0.87 (0.57, 1.32) |
| **During pregnancy, you can get regular exercise** | |  |
|  | n | 965 |
|  | *p* value | 0.407 |
|  | Unsure or very unsure | 0.78 (0.47, 1.29) |
|  | Neither unsure or sure | 1.0 (reference) |
|  | Sure or very sure | 0.77 (0.52, 1.13) |
| **I control my emotions by not expressing them** | |  |
|  | n | 968 |
|  | *p* value | 0.561 |
|  | Almost never or sometimes | 1.03 (0.64, 1.66) |
|  | About half the time | 1.0 (reference) |
|  | Most of the time or almost always | 0.85 (0.49, 1.48) |
| **When I am upset, I have difficulty controlling my behaviour** | |  |
|  | n | 958 |
|  | *p* value | 0.965 |
|  | Almost never or sometimes | 1.02 (0.60, 1.73) |
|  | About half the time | 1.0 (reference) |
|  | Most of the time or almost always | 0.96 (0.50, 1.86) |
| **When I’m upset, it takes me a long time to feel better** | |  |
|  | n | 958 |
|  | *p* value | 0.323 |
|  | Almost never or sometimes | 0.71 (0.43, 1.17) |
|  | About half the time | 1.0 (reference) |
|  | Most of the time or almost always | 0.64 (0.36, 1.16) |
| **When I’m upset, I believe that there’s nothing I can do to make myself feel better** | |  |
|  | n | 967 |
|  | *p* value | 0.903 |
|  | Almost never or sometimes | 1.25 (0.46, 3.35) |
|  | About half the time | 1.0 (reference) |
|  | Most of the time or almost always | 1.20 (0.39, 3.66) |
| **When I'm upset, I know I can find a way to eventually feel better** | |  |
|  | n | 965 |
|  | *p* value | 0.671 |
|  | Most of the time or almost always | 0.91 (0.58, 1.40) |
|  | About half the time | 1.0 (reference) |
|  | Almost never or sometimes | 1.09 (0.60, 1.99) |
| **When I am upset, I become embarrassed for feeling that way** | |  |
|  | n | 965 |
|  | *p* value | 0.955 |
|  | Almost never or sometimes | 1.00 (0.62, 1.60) |
|  | About half the time | 1.0 (reference) |
|  | Most of the time or almost always | 1.06 (0.58, 1.93) |
| **I feel that I must do things perfectly or not do them at all** | |  |
|  | n | 964 |
|  | *p* value | 0.522 |
|  | Almost never or sometimes | 0.91 (0.62, 1.33) |
|  | About half the time | 1.0 (reference) |
|  | Most of the time or almost always | 1.13 (0.68, 1.86) |
| **During the 3 months before pregnancy, how often were you dieting?** | |  |
|  | n | 968 |
|  | *p* value | 0.002 |
|  | Never or rarely | 1.0 (reference) |
|  | Often or always | 1.70 (1.22, 2.36) |
| **During the 3 months before pregnancy, how often did you limit your carbohydrate and sugar intake?** | |  |
|  | n | 969 |
|  | *p* value | 0.024 |
|  | Never or rarely | 1.0 (reference) |
|  | Often or always | 1.35 (1.04, 1.74) |
| **During the 3 months before pregnancy, did you have feelings of guilt after overeating?** | |  |
|  | n | 969 |
|  | *p* value | 0.008 |
|  | Never or rarely | 1.0 (reference) |
|  | Often or always | 1.45 (1.10, 1.91) |
| **During the 3 months before pregnancy, did you ever feel that when you started eating you just couldn’t stop?** | |  |
|  | n | 968 |
|  | *p* value | 0.123 |
|  | Never or rarely | 1.0 (reference) |
|  | Often or always | 1.39 (0.91, 2.11) |
| **During the 3 months before pregnancy, did you want to eat when you were emotionally upset?** | |  |
|  | n | 968 |
|  | *p* value | <0.0001 |
|  | Never or rarely | 1.0 (reference) |
|  | Often or always | 1.67 (1.25, 2.23) |
| **I eat sensibly when with others, but overdo so when I’m alone** | |  |
|  | n | 965 |
|  | *p* value | <0.0001 |
|  | No | 1.0 (reference) |
|  | Yes | 1.61 (1.24, 2.08) |
| **If others saw how much I ate, then I’d feel ashamed** | |  |
|  | n | 964 |
|  | *p* value | 0.003 |
|  | No | 1.0 (reference) |
|  | Yes | 1.62 (1.18, 2.22) |
| **Overeating justification- I will eat healthier later** | |  |
|  | n | 961 |
|  | *p* value | 0.017 |
|  | Never | 1.0 (reference) |
|  | Rarely, frequently, or always | 1.45 (1.07, 1.96) |
| **Overeating justification- I will compensate by eating less later** | |  |
|  | n | 959 |
|  | *p* value | 0.476 |
|  | Never | 1.0 (reference) |
|  | Rarely, frequently, or always | 1.10 (0.85, 1.42) |
| **Overeating justification- I will compensate by doing some exercise later** | |  |
|  | n | 962 |
|  | *p* value | 0.035 |
|  | Never | 1.0 (reference) |
|  | Rarely, frequently, or always | 1.37 (1.02, 1.83) |
| **I have a lot of fear regarding the health of my baby** | |  |
|  | n | 950 |
|  | *p* value | 0.102 |
|  | No | 1.0 (reference) |
|  | Yes | 1.28 (0.95, 1.73) |
| **Nausea related to pregnancy** | |  |
|  | n | 967 |
|  | *p* value | 0.733 |
|  | Never or 1time/week | 1.0 (reference) |
|  | ≥1 time/day | 1.05 (0.79, 1.41) |
| **Food cravings related to pregnancy** | |  |
|  | n | 952 |
|  | *p* value | 0.543 |
|  | Never or 1time/week | 1.0 (reference) |
|  | ≥1 time/day | 1.09 (0.83, 1.42) |
| **Eat something to cope with nausea** | |  |
|  | n | 970 |
|  | *p* value | 0.455 |
|  | No | 1.0 (reference) |
|  | Yes | 0.91 (0.70, 1.17) |
| **Avoiding eating to cope with nausea** | |  |
|  | n | 970 |
|  | *p* value | 0.153 |
|  | No | 1.0 (reference) |
|  | Yes | 0.79 (0.58, 1.09) |
| **Take medication to cope with nausea** | |  |
|  | n | 970 |
|  | *p* value | 0.206 |
|  | None | 1.0 (reference) |
|  | Medication use | 1.16 (0.69, 1.95) |
|  | Other ways | 0.82 (0.60, 1.13) |
| **Eat what I crave to cope with cravings** | |  |
|  | n | 970 |
|  | *p* value | 0.816 |
|  | No | 1.0 (reference) |
|  | Yes | 1.03 (0.78, 1.36) |
| **Avoid what I crave to cope with cravings** | |  |
|  | n | 970 |
|  | *p* value | 0.916 |
|  | No | 1.0 (reference) |
|  | Yes | 0.98 (0.67, 1.43) |
| **Distract myself to cope with cravings** | |  |
|  | n | 970 |
|  | *p* value | 0.584 |
|  | No | 1.0 (reference) |
|  | Yes | 1.09 (0.79, 1.51) |
| **Sleep** | |  |
|  | n | 961 |
|  | *p* value | 0.435 |
|  | < 8 hr/day | 1.0 (reference) |
|  | ≥ 8 hr/day | 1.11 (0.86, 1.43) |
| **Total physical activity** | |  |
|  | n | 894 |
|  | *Pp* value | 0.475 |
|  | Low | 1.0 (reference) |
|  | Medium | 1.21 (0.87, 1.67) |
|  | High | 1.03 (0.75, 1.43) |
| **Preferred body size image before pregnancy** | |  |
|  | n | 965 |
|  | *p* value | 0.009 |
|  | Underweight | 0.59 (0.42, 0.82) |
|  | Normal weight | 1.0 (reference) |
|  | Overweight or obese | 0.89 (0.54, 1.47) |
| **Comparison between perceived and preferred body size image** | |  |
|  | n | 965 |
|  | *Pp* value | 0.006 |
|  | Actually smaller than preferred | 0.78 (0.42, 1.47) |
|  | Accurate | 1.0 (reference) |
|  | Actually larger than preferred | 1.47 (1.11, 1.94) |
| **Comparison between BMI and perceived body size image** | |  |
|  | n | 966 |
|  | *p* value | <0.0001 |
|  | Actually smaller than perceived | 0.69 (0.33, 1.43) |
|  | Accurate | 1.0 (reference) |
|  | Actually larger than perceived | 1.81 (1.39, 2.37) |
| **Sitting time/day, hr** | |  |
|  | n | 894 |
|  | *p* value | 0.465 |
|  | Continuous variable | 1.02 (0.97, 1.06) |
| **TPB score** | |  |
|  | n | 963 |
|  | *Pp* value | 0.808 |
|  | Continuous variable | 0.98 (0.86, 1.12) |
| **Personality- Extraversion** | |  |
|  | n | 952 |
|  | *Pp* value | 0.732 |
|  | Continuous variable | 1.02 (0.93, 1.11) |
| **Personality- Agreeableness** | |  |
|  | n | 945 |
|  | *p* value | 0.052 |
|  | Continuous variable | 1.14 (1.00, 1.30) |
| **Personality- Conscientiousness** | |  |
|  | n | 951 |
|  | *p* value | 0.019 |
|  | Continuous variable | 0.86 (0.76, 0.98) |
| **Personality- Emotional Stability** | |  |
|  | n | 953 |
|  | *Pp* value | 0.537 |
|  | Continuous variable | 0.97 (0.88, 1.07) |
| **Personality- Openness** | |  |
|  | n | 956 |
|  | *p* value | 0.945 |
|  | Continuous variable | 1.00 (0.89, 1.13) |

BMI, body mass index; HCP, health care provider; TPB, theory of planned behavior.
