# Supplementary material for: Prediction of excess pregnancy weight gain using psychological, physical, and social predictors: A validated model in a prospective cohort study
Source: PLoS One. 2020 Jun 2;15(6):e0233774. doi: 10.1371/journal.pone.0233774 (PMC7266315; doi:10.1371/journal.pone.0233774)
Supplement: S6 File — Sensitivity analyses using samples restricted to A) women with age > 19 years old and B) women without inadequate weight gain. (DOCX) [file pone.0233774.s006.docx]

**S6 File. Sensitivity analyses using samples restricted to A) women with age > 19 years old and B) women without inadequate weight gain.**

| **Sensitivity Analysis Table A1.** **Factors which predict excess pregnancy weight gain among women aged 20 years and older (n=956)** | | |
| --- | --- | --- |
| **Predictors** | **aOR (95% CI)** | ***p* value** |
| **Parity** |  |  |
| Nulliparous | 1.75 (1.22 to 2.51) | 0.003 |
| Multiparous | 1.0 (reference) | NA |
| **Prepregnancy BMI** |  |  |
| Underweight | 0.30 (0.10 to 0.89) | 0.030 |
| Normal weight | 1.0 (reference) | NA |
| Overweight | 3.23 (2.01 to 5.21) | <0.001 |
| Obese | 0.99 (0.58 to 1.66) | 0.959 |
| **Frequency of eating in front of a screen** |  |  |
| None or almost no meals | 1.0 (reference) | NA |
| Some meals | 1.73 (1.17 to 2.57) | 0.006 |
| Most meals or more | 1.89 (1.12 to 3.18) | 0.016 |
| **Planned pregnancy weight gain** |  |  |
| Not reported | 1.35 (0.66 to 2.72) | 0.410 |
| Within guidelines | 1.0 (reference) | NA |
| Below guidelines | 0.80 (0.52 to 1.25) | 0.327 |
| Above guidelines | 2.88 (1.76 to 4.71) | <0.001 |
| **I control my emotions by not expressing them** |  |  |
| Most of the time or almost always | 1.0 (reference) | NA |
| About half the time | 0.58 (0.36 to 0.95) | 0.029 |
| Almost never or sometimes | 0.51 (0.26 to 0.99) | 0.047 |
| **Conscientious personality** | 0.84 (0.70 to 1.00) | 0.046 |
| Legend: aOR, adjusted odds ratio; CI, confidence interval; NA, not applicable; BMI, body mass index. | | |

| **Sensitivity Analysis Table A2.** **Areas under the receiver operating characteristic curves derived from the training and testing samples among women aged 20 years and older** | | |
| --- | --- | --- |
|  | **AUC** | **p for differences** |
| Training sample (n=637) | 0.73 (0.70 to 0.77) | 0.057 |
| Testing sample (n=319) | 0.66 (0.60 to 0.73) |  |

| **Sensitivity Analysis Table B1.** **Factors which predict excess pregnancy weight gain using adequate weight gain as the reference (n=803)** | | |
| --- | --- | --- |
| **Predictors** | **aOR (95% CI)** | ***p* value** |
| **Parity** |  |  |
| Nulliparous | 1.53 (1.01 to 2.31) | 0.045 |
| Multiparous | 1.0 (reference) | NA |
| **Prepregnancy BMI** |  |  |
| Underweight | 0.30 (0.10 to 0.93) | 0.037 |
| Normal weight | 1.0 (reference) | NA |
| Overweight | 2.03 (1.17 to 3.52) | 0.012 |
| Obese | 1.43 (0.79 to 2.57) | 0.236 |
| **Frequency of eating in front of a screen** |  |  |
| None or almost no meals | 1.0 (reference) | NA |
| Some meals | 2.08 (1.33 to 3.27) | 0.001 |
| Most meals or more | 2.62 (1.38 to 4.98) | 0.003 |
| **Planned pregnancy weight gain** |  |  |
| Not reported | 2.55 (0.94 to 6.92) | 0.066 |
| Within guidelines | 1.0 (reference) | NA |
| Below guidelines | 1.16 (0.69 to 1.95) | 0.583 |
| Above guidelines | 2.88 (1.69 to 4.91) | <0.001 |
| **During the three months before pregnant, did you have feelings of guilt after overeating?** |  |  |
| Never or rarely | 1.0 (reference) | NA |
| Often or always | 1.66 (1.06 to 2.60) | 0.027 |
| **Think that family and friends believe that pregnant women should not exert themselves physically** |  |  |
| Disagree or strongly disagree | 1.34 (0.74 to 2.42) | 0.330 |
| Neither disagree nor agree | 1.0 (reference) | NA |
| Agree or strongly agree | 0.54 (0.31 to 0.94) | 0.029 |
| **Think that family and friends believe that pregnant women should not be worried about gaining too much weight during pregnancy** |  |  |
| Disagree or strongly disagree | 1.15 (0.67 to 1.97) | 0.603 |
| Neither disagree nor agree | 1.0 (reference) | NA |
| Agree or strongly agree | 2.26 (1.24 to 4.10) | 0.008 |
| Legend: aOR, adjusted odds ratio; CI, confidence interval; NA, not applicable; BMI, body mass index. | | |

| **Sensitivity Analysis Table B2.** **Areas under the receiver operating characteristic curves derived from the training and testing samples using adequate weight gain as the reference** | | |
| --- | --- | --- |
|  | **AUC** | **p for differences** |
| Training sample (n=535) | 0.74 (0.69 to 0.78) | 0.047 |
| Testing sample (n=268) | 0.65 (0.59 to 0.72) |  |
