# Supplementary material for: Prediction of excess pregnancy weight gain using psychological, physical, and social predictors: A validated model in a prospective cohort study
Source: PLoS One. 2020 Jun 2;15(6):e0233774. doi: 10.1371/journal.pone.0233774 (PMC7266315; doi:10.1371/journal.pone.0233774)
Supplement: S7 File — (DOCX) [file pone.0233774.s007.docx]

**Literature Search for Prediction Model for GWG**

Table of Contents

[**Search Objective** 1](#_Toc13512922)

[**Search Results:** 1](#_Toc13512923)

[**OVID Search Strategy:** 1](#_Toc13512924)

[References 2](#_Toc13512925)

**Search Objective**:

To find any existing **prediction models** for excess GWG (gestational weight gain or pregnancy weight gain) above IOM guidelines with **validation**.

**Search Results:**

Our search yielded 3 prediction models.^1,2,3^ **Thomas 2012 was the only validated model but modeled the “impact of changes in dietary energy intake on GWG” using *pre*-pregnancy dual-energy X-ray absorptiometry scans.**^1^ Two studies used weight measurements taken early in pregnancy to predict GWG^2,3^. Chmitroz 2012 measured weight each trimester^2^ and Knabl 2014 used 4-week intervals^3^. All of the studies developed separate models for different BMI categories. However, training & testing/validation were not mentioned in Chmitroz 2012 & Knabl 2014, so they are not validated. Lastly, Banjari 2015 used cluster analysis to find predictors of GWG but is NOT a prediction model as it lacks validation and training data.^4^

# **OVID Search Strategy:**

Date: 2018-November-19

| **#** | **Searches** | **Results** |
| --- | --- | --- |
| 1 | exp pregnancy/ and exp weight gain/ | 3992 |
| 2 | (pregnan: adj4 weight gain).mp. | 2053 |
| 3 | (gestation: adj2 weight gain).mp. | 1840 |
| 4 | pregnant women/ and exp weight gain/ | 120 |
| 5 | exp pregnancy complications/ and exp weight gain/ | 2146 |
| 6 | (1 or 2 or 4 or 5) and 3 | 1443 |
| 7 | Predictive Value of Tests/ | 169451 |
| 8 | (predic: adj1 model:).mp. | 41741 |
| 9 | (predic: adj1 tool:).mp. | 3881 |
| 10 | valid:.mp. | 501101 |
| 11 | (training adj1 data:).mp. | 3422 |
| 12 | 7 or 8 or 9 or 10 or 11 | 683570 |
| 13 | 6 and 12 | 64 |

# References

1. Thomas DM, Navarro-Barrientos JE, Rivera DE, Heymsfield SB, Bredlau C, Redman LM, Martin CM, Lederman SA, Collins LM, Butte NF. Dynamic energy-balance model predicting gestational weight gain. Am. J. Clin. Nutr. 2012 Jan;95(1):115-122

<https://academic.oup.com/ajcn/article/95/1/115/4576495>

1. Chmitroz A, von Kries R, Rasmussen KM, Nehring I, Ensenauer R. Do trimester-specific cutoffs predict whether women ultimately stay within the Institute of Medicine/National Research Council guidelines for gestational weight gain? Findings of a retrospective cohort study. Am J Clin Nutr. 2012 Jun;95(6):1432-7

<https://academic.oup.com/ajcn/article/95/6/1432/4568392>

1. Knabl J, Riedel C, Gmach J, Ensenauer R, Brandlhuber L, Rasmussen KM, Schiessl B, von Kries R. Prediction of excessive gestational weight from week-specific cutoff values: a cohort study. J Perinatol. 2014 May;34(5):351-6

<https://www.nature.com/articles/jp201422>

1. Banjari I, Kenjeric D, Solic K, Mandic ML. Cluster Analysis as a Prediction Tool for Pregnancy Outcomes. Coll. Antropol. 2015;39(1):247-252

<http://www.collantropol.hr/antropo/article/view/1230/1133>
