# Supplementary material for: Prediction of excess pregnancy weight gain using psychological, physical, and social predictors: A validated model in a prospective cohort study
Source: PLoS One. 2020 Jun 2;15(6):e0233774. doi: 10.1371/journal.pone.0233774 (PMC7266315; doi:10.1371/journal.pone.0233774)
Supplement: S8 File — (DOCX) [file pone.0233774.s008.docx]

**1.0 PROBLEM STATEMENT**

Half^1;2^ of women, or more^3;4^, exceed the 2009 national guidelines^5;6^ for weight gain during pregnancy, significantly increasing the risks to themselves^7-11^ and their infants^12-14^. Specifically, excess pregnancy weight gain increases maternal risks for high blood pressure^8^, diabetes^7^, cesarean section^7^, postpartum weight retention^15-17^ and obesity^11^. Excess pregnancy weight gain is a key risk factor for high infant birth weight^12;13^ which in turn, is associated with being overweight^18^ and obese during adolescence and adulthood^19^. Hence, **pregnancy represents a critical period for women and their infants that may negatively alter their weight trajectories towards obesity**.

Despite this fact, interventions to prevent excess weight gain during pregnancy have been largely unsuccessful^20-22^ and a recent systematic review concluded that the format and intensity of effective interventions remains uncertain^23^. Given the significant consequences of excess weight gain and the lack of success of existing interventions, a broader understanding of pregnancy weight gain has been called for. Multiple recent meta-analyses of successful interventions have highlighted a need for more study of psychological factors influencing weight gain^24-26^, e.g. binge eating and motivation. In response, we undertook a systematic review of psychological factors associated with excess pregnancy weight gain^27^, a cross-sectional survey^28^ and a pilot prospective cohort study^29^, all of which guided the development of this proposal.

**2.0 Objectives**

**Quantitative prospective cohort study:**

Primary objectives: To understand the relative importance of psychological and other determinants of total pregnancy weight gain in excess of the recommendations in the national guideline, termed excess pregnancy weight gain. (Figure 1, p.2)

**3.0 Research Question**

**Quantitative prospective cohort study:**

1. Which psychological and other factors during early gestation (8-20 weeks) predict excess weight gain during pregnancy, as defined by the current national guideline using a validated prediction model?

**Figure 1 Overview and *justification* of Study 1 exposures and outcome** Since our study is ***exploratory***, we are not testing an existing model but instead, are guided by elements from established psychological theories of motivation, for example *Theory of Planned Behavior (TPB)*^30^ from which we include assessment of i) *control beliefs* e.g. self-efficacy and locus of control, ii) *normative beliefs* e.g. family and friends’ beliefs and iii) *outcome beliefs* e.g. perceived risks of excess gain. However, we and others in the weight field^31^ recognize that **reliance on any single, existing model at this stage of our understanding of pregnancy weight gain would be too narrow** (for instance, a recent meta-analysis noted *TPB* accounted for 27% and 39% of variance in behavior and intention^32^, respectively, and 34% of the variance of health behaviors^33;34^). Hence, we also draw on constructs from *pregnancy literature^27^, “Disordered Eating” literature [includes Dietary Restraint Theory and overeating* *(binge eating, etc)*^35-38^ as well as the *eating disorders literature (impulsivity, emotional suppression, etc)]*^39^. This figure represents our interest in understanding, at this exploratory stage, the relative contribution of modifiable and non-modifiable determinants impacting the clinical phenomenon of excess pregnancy weight gain (not necessarily suggesting a causal path):

*DETERMINANTS*

*Outcome*

1) **COGNITION** (e.g. *control*, *normative &* *outcome beliefs***;** *PREGNANCY LIT* (*target weight gain, risks in pregnancy with excess gain*, etc.)

2) **PERSONALITY** (e.g. *impulsivity*, etc)

3) **AFFECT** (e.g*. emotional eating*, etc)

1) *Disordered eating* *(e.g. dietary restraint, binge eating, night eating)*

2) Other health & risk behaviours (e.g. smoking, diet, activity, sleep, etc)

1) Physiologic (e.g. prepregnancy BMI, increased blood & fat, 1^st^ pregnancy, etc)

2) Pathologic factors (e.g. edema with hypertension, etc.)

(E.g. age, education, income, etc)

**BEHAVIOURAL**

**PSYCHOLOGICAL**

**PHYSICAL**

*(Not modifiable in preg.)*

EXCESS **PREGNANCY WEIGHT GAIN**

**SOCIO-DEMOGRAPHIC**

*(Not modifiable in preg.)*

**4.0 Background**

***4.1 National pregnancy weight guidelines were released to try to minimize maternal and infant risks***

In the midst of rising rates of pregnancy weight gain, guidelines were released in 2009 by the Institutes of Medicine^5^ and in 2010 by Health Canada^6^. Although the previous guidelines had focused on reducing infant complications from inadequate gain, the most recent version also considered excess gain. Women with higher pre-pregnancy body mass indices (BMI) require less weight gain.

Table 1 National guidelines for pregnancy weight gain according to pre-pregnancy BMI^5;6^

| Pre-pregnancy body mass indices (BMI) | Recommended total weight gain |
| --- | --- |
| Underweight (BMI <18.5 kg/m^2^) | 12.5-18 kg |
| Normal weight (BMI 18.5-24.9 kg/m^2^) | 11.5-16 kg |
| Overweight (BMI 25-29.9 kg/m^2^) | 7-11.5 kg |
| Obese (BMI > 30 kg/m^2^) | 5-9 kg |

***4.2 The clinical problem: Excess pregnancy weight gain occurs in the majority of women and is associated with increased risks to maternal and infant health both during and after pregnancy***

Approximately **50**^1;2^**-60%**^3;4^ **of pregnant women gain in excess of the guidelines**^5;6^, consistent with our local data^29^. Moreover, the proportion of women gaining in excess is increasing^40^.

During pregnancy, excess weight gain is associated with maternal risks, including a doubling of the risk of preeclampsia (high blood pressure which affects the kidneys, liver or hematologic system)^8^. Excess pregnancy gain increases risks of diabetes, forceps/vacuum assisted birth or cesarean section^7^.

**High pregnancy weight gain correlates with both short- and long-term maternal weight retention**^15-17^ and increasing BMI^9^. Excess pregnancy weight gain is associated with a 2- to 3-fold increased risk of becoming overweight after delivery^41^ and is an important predictor of obesity in midlife^11^. *Women of childbearing age are particularly vulnerable to obesity* as they are at greatest risk of major weight gain. **Many obese women attribute their weight gain to childbearing**^42^.

High pregnancy weight gain is a key risk factor for *high birth weight*^12;13;43^. Immediate risks of high birth weight include increased risks of *trauma at birth*^44;45^, including potential entrapment of the infant’s shoulders (shoulder dystocia)^46^, injury to the nerves innervating the arm (brachial plexus injury) and most importantly, asphyxia^47^. Long term risks of high birth weight include a **doubling of the risk of offspring being overweight**^18^ **or obese**^19^ in childhood and adulthood. In utero, excess nutrients alter the development of fat cells, resulting in a permanent increase in the capacity to form new cells /store fat^48^. Hence, **pregnancy is a critical period for both women and infants that may negatively alter their weight trajectories, contributing to the obesity epidemic.**

Prevention of obesity is key, given challenges with treatment, and given the importance of the problem. Obesity is one of Canada’s dominant and most costly health problems^49^. Almost 6 out of 10 Canadian adults are overweight or obese^50^. *Obesity is the second leading cause of preventable death*^51^, with increases in cardiovascular disease, diabetes, some cancers^52^ and depression^53^. The World Health Organization calls obesity one of the most obvious, yet neglected, global health problems^54^.

***4.3 Excess pregnancy weight gain is associated with both non-modifiable and modifiable factors***

Established risk factors for excess pregnancy weight gain include ones that are **not modifiable** during pregnancy (physiologic ones e.g. high BMI^55;56^, first pregnancy^55^; sociodemographic ones e.g. young age^57^, low income or education^58^) and **potentially modifiable** ones which are psychologic in nature (cognition: e.g. planned or target weight gain above guidelines^29^) or behaviour: e.g. physical activity^55^ and diet^55^). More psychological factors are detailed in Section 4.5.

***4.4 Many interventions have been unsuccessful in preventing excess pregnancy weight gain, resulting in calls to understand psychological underpinnings***

Systematic reviews have concluded that effective interventions to limit weight gain in pregnancy are not yet established^23;59;60^, a finding repeated in other systematic reviews which found no improvement in the proportion of women exceeding the guidelines. Although some resource intensive interventions^63-65^ are successful in subgroups of women (with obesity or low incomes^66^), other subgroups show no benefit^20-22^, and alarmingly, others show trends toward harm (increasing excess gain^67^). As intervention trials have met with limited success, *researchers have called for a broader understanding of pregnancy weight gain*^68^.

**Multiple recent meta-analyses have highlighted a need for more study of psychological factors influencing pregnancy weight gain**, recommending that future studies appreciate that the etiology of excess pregnancy weight gain may include psychological factors^24-26^ e.g. knowledge, body image, coping skills and support, etc.^26^. An appreciation of the psychological etiology of excess weight gain requires a focus on psychological mechanisms underpinning behavioural change. These *antecedents* of behavioral modification have previously not been assessed in interventions which may help explain why behavioral interventions to prevent excessive pregnancy weight gain have overall been ineffective^25^. This recent literature recommends targeting psychological factors which might impede behavioural change, such as self-efficacy, body image and motivation^25^. This call for study of psychological influences extends outside of pregnancy in the literature on obesity prevention and intervention, which has appealed for study of personality^69^.

***4.5 Responding to the call for more study of psychological determinants of pregnancy weight gain, the foundational work completed for this proposal includes a systematic review which identified* *psychological factors requiring research and a pilot study demonstrating the feasibility and interest of the research concept and approach*** *(Justification for included determinants)*

Our CIHR-funded systematic review of the literature^27^revealed that some psychological factors were associated with excess pregnancy weight gain, in terms of cognition (e.g. weight locus of control^70^, target weight gain^71^, weight attitudes^72^ , barriers to healty eating^70^), and behaviour (e.g. dietary restraint^73^). While suggesting *some useful predictors*, most studies were underpowered and none validated a predictive model as we will.

Our systematic review revealed that in general affect, including stress, was not associated with excess gain^27^. However, pregnancy-related anxiety had not been studied, *therefore, we will include it*.

Our systematic review noted a number of other potentially important psychological factors that had not been explored, including aspects of personality and behaviour, and hence we targeted some of these in our pilot cross-sectional study^28^ and then in our pilot prospective cohort study^29^. As detailed in the appended paper, we found that predictors of excess pregnancy weight gain included a history of binge eating and planned weight gain above the guidelines (adj RR 6.51, 95% CI 1.03-41.18 and adj RR 9.44, 95% CI 2.64-33.80, respectively.*We have begun to uncover key constructs and a number of related eating behaviours, below in Section 4.6, require study.*

Our pilot study guided selection of determinants (e.g. removing global constructs such as self-esteem, which were not associated with weight gain, in favour of specific ones such as weight locus of control) but also demonstrated *feasibility*: all clinics (100%) and 90% of approached women agreed to participate and 80% of women overall participated. Since only 11% were enrolled <21 weeks, our proposed recruitment is now appropriately long. Final pregnancy weight was available for 97%, hence our sample size was minimally increased to accommodate this missing primary outcome data.

***4.6 Lessons from non-pregnant literature: Disordered Eating is common with excess weight, and stratification by weight class is key to this understanding*** *(Justification for included determinants)*

Disordered Eating, an umbrella term, includes not only the *Eating Disorder* literature*,* from which we will draw constructs such as *impulsivity^74^ and emotional supression^75^,* but also the much more common ‘*problem eating’*^76^ i.e. *restrained eating* (dieting), *binge eating* and *night eating*^[[1]](#footnote-1)^ ^35;37^*.*

A milestone multisite study of almost two thousand people found that *19% of community controls exhibited episodic overeating, and a further 6% also exhibited loss of control*, moreover, *30%* of attendees at *hospital* weight control programs met criteria for a formal diagnosis of *binge eating disorder*^38^. This landmark paper *emphasized the importance of stratification according to excess vs normal weight weight* to reflect the difference in the proportions of people with Disordered Eating^38^. Stratification by weight category is now standard in the Disordered Eating literature^80-82^. Disordered Eating is ameliorable to intervention; cognitive behavioral interventions such as psychotherapy resulted in *large* reductions in binge eating^83^. *We will address the need for more study of Disordered Eating which, given the prevalence in the non-pregnant overweight/obesity literature, is anticipated to play an key role in excess pregnancy weight gain in overweight/obese women*.

***4.7 A final reason from the non-pregnant literature adds to the justification to study psychological factors: tailoring treatment to psychological traits improves health behaviours like diet & weight***

Although our main focus in this proposal is to understand psychological and other determinants of weight gain, *future* work will use these results to design effective interventions. Rather than delivering the same intervention to all, tailoring uses patient characteristics to target the content of the interventions^92^. *Tailoring promotes behaviour change by providing personally relevant feedback.* Hence, tailoring mimics the clinical scenario of interpersonal counselling^93;94^. Examples of successful tailored interventions include increasing *self-efficacy* regarding exercise^95^, and matching weight reduction interventions to *locus of control* beliefs (a self-directed program for “internals” and a group program for “externals” resulted in a tendency to lose more weight than when the programs were "mismatched")^96^. In another weight intervention, *programs designed to increase self-efficacy were successful with women who identify an internal locus of control but not an external one*^97^. Computer tailoring enables valid self-assessment and comparison to recommendations or peer group^93^, key components of the Theory of Planned Behaviour which is the model underpinning this proposal.

A *36% increase in health behaviours with tailored interventions* was found in a recent meta-analysis^92^. Another meta-analysis demonstrated that interventions focused on preventative behaviors and screening have been the most successful applications of tailoring^98;99^. Tailoring is particularly useful for complex behaviours, such as dietary ones^94^. Furthermore, systematic reviews have demonstrated tailoring’s promise with computer-tailored^92^ and internet-based interventions^100^.

***4.8 Summary of the importance of the topic and guidance from our pilot data***

Excess pregnancy weight gain increases maternal and infant risks during and after pregnancy, including obesity, one of Canada’s dominant health problems. Most pregnant women now gain in excess of the guidelines. **Given the major risks to both mothers and infants, there is an urgent need to understand the determinants of pregnancy weight gain**, as the first step in developing innovative interventions to promote healthy weight gain. We *uniquely* approach this question, guided by our pilot data from women <28 weeks which now needs to be studied*: 1) earlier in gestation* (<20 weeks, so future interventions could be implemented to prevent excess gain)*, 2) comprehensively assessing* psychological and other factors including ones noted by our systematic review as being unexplored and ones from the non-pregnant literature, e.g.‘binge eating’, 3) with a predictive model that will be validated.

**5.0 Research** **APPROACH**

**5.1 Study Design**

To understand the psychological and other determinants of excess pregnancy weight gain, we propose a **prospective cohort study**.

**5.2 Study 1: Multi-centre prospective cohort study**

**5.2.1 Study Setting**

We propose the province of *Ontario* for this research since: i) it has the largest proportion of births in Canada (140,135 of a total 377,636 in 2011^101^) and ii) we have better *feasibility* due to closer contact with collaborating centres, including personal relationships or histories of successful recruitment. The sites will include large and smaller urban centres from the five regions of Ontario^102^: Ottawa (East), Toronto (considered its own region), Burlington (Central), Hamilton & Brantford (West), and Thunder Bay (North). We will include clinics from the main groups of pregnancy health care providers: *obstetricians, family physicians and midwives*^103^.

**5.2.2. Population inclusion criteria** will be pregnant women with a live, singleton fetus from 8 weeks + 0 days to 20 weeks + 6 days gestation who can read/write English well enough to complete the survey and plan to give birth at the same centre as they were recruited, to facilitate outcome collection. [Justification: i) The lower limit (8 weeks) was selected as after that the miscarriage rate point is low (0.5%^104^ -3.0%^105^). ii) Twenty weeks, half way through a term pregnancy, was chosen as a balance between being early enough in gestation that future interventions could target this period with a reasonable opportunity for improving pregnancy weight gain and yet being feasible for recruitment, as shown by our pilot study^29^. iii) We will begin by studying English-literate women who make up the majority of pregnant women in Ontario. (Future research will involve other groups.)]

**5.2.3 Population exclusion criteria** will be for the following (rare) conditions: i) *twins* or higher order multiples (2-3% of pregnancies) since weight gain recommendations differ^5;6^, ii) a fetus with a known lethal *anomaly* (~0.0023%^106^) or a termination of pregnancy (0.5%^106^) after enrollment, and iii) women with *pathologies that severely impact weight gain* (due to extreme diets e.g. bariatric surgery^107^, anorexia^108;109^, and bulimia^108^, which together constitute much less than 1% of pregnant women).

**5.2.4 Recruitment and study flow**

We will attempt to recruit *consecutive* women in *early pregnancy* between 8-20 weeks, *to inform future interventions* to optimize weight gain. Our pilot study suggested recruitment would be close to 20 weeks for the majority of women and participation would be ~80%^29^. Interested women will meet with research staff in the clinics. Written, informed consent will be obtained.

**5.2.6 Data Collection** will occur using:i) questionnaires and ii) antenatal records and hospital charts.

**i)** The **Main Questionnaire** (which will be administered at ~12-20 weeks, and a 2^ndary^ one at ~ 32 weeks) will measure our **exposures**: **psychological and other determinants** of excess pregnancy weight gain. Selection of psychological variables was based on our systematic review of the literature & our pilot work^29^ (Section 4.5) and non-pregnant literature on Disordered Eating (Section 4.6).

**Informed by our systematic review** of PSYCHOLOGICAL DETERMINANTS of excess pregnancy weight gain^27^ factors felt to be important by the team will be included:

| 1. Construct | **Previously explored factors in our systematic review** | **Previously *un*explored factors in pregnancy** |
| --- | --- | --- |
| Cognition | *Weight locus of control*^110a^, *target weight gain*^71a^, *weight attitudes*^110a^,  *body image*^111a^, *Barriers to Healthy Eating*^112a^  self-efficacy^110a^ | *Nutrition knowledge*^113b^, *normative factors*^c^ |
| Affect | No association between general affect & pregnancy weight gain | *Pregnancy-related anxiety*^114^ |
| Personality | Not adequately studied | *Impulse control*^74d^, *perfectionism*^115d^, *motivation*^116d^,*expressive suppression*^75d^ & *Big 5 Factors*^117^ |
| Behaviour | *Dietary restraint*^118b^*;* Other behaviours: *diet*^119b^, *physical activity*^120,121b^, *sleep*^b^, *smoking*^b^, *eating in front of a screen*^b^ | *Binge Eating Scale*^122e^, *night eating*^e^*, emotional eating*^39e^ |

Legend & Justification: ^a^=associated with excess gain in our systematic review; ^b^=based obesity literature; ^c^=investigator developed based on TPB; ^d^=based on eating disorder literature, ^e^=based on other disordered eating literature.

Informed by the pregnancy literature (Section 4.3), we will collect information on SOCIODEMOGRAPHIC DETERMINANTS (including maternal *age*^57^, education, income) and PHYSICAL DETERMINANTS of excess pregnancy weight gain, including *BMI* and the *number of pregnancies* (overweight women are more likely to gain in excess^123^ as are first time mothers^2^).

**5.2.7 Questionnaire development.** The questionnaire has been assessed for **content validity** by five individuals with clinical expertise to ensure that determinants of excess pregnancy weight gain are captured. We used existing, reliable scales wherever possible, with the development of a few items to accomplish our objectives. Additionally, they will be *pilot tested* in a sample of 8 pregnant women. They will be asked to complete the questionnaire using a “think aloud” approach to assess *readability*, comprehensibility and reactions to questions, with revision as appropriate. Pretesting will ensure that items are clear and interpreted correctly^124^.

**5.2.8 Questionnaire administration** will be offered on hard copy. The main part of the questionnaire takes approximately 15-20 minutes, and hence most women will likely complete it in the clinic (anticipated **95%**, based on our pilot prospective cohort study^29^. For extensive scales which might have jeopardized completion, we sought shorter, reliable ones or reliable subscales.)

We anticipate *> 85% response rate*, since we will: 1) use a modified **Dillman** approach^125^, of up to three attempts to contact the participant (*without calling,* our team achieved a 74% response rate during pregnancy^126^), 2) offer to complete the questionnaire over the phone which increases response rate above that of a telephone reminder^127^, 3) visibly locate the research staff in the clinics where the women will see them when they return for clinical visits and 4) provide *an incentive*. ib) At ~32 weeks, as per participant preference, we will offer email or hard copy of the 2^ndary^ Questionnaire. It will contain the same questions as the Main Questionnaire, apart from non-mutable ones (e.g. age, prepregnancy height, etc). The Dillman approach with the same *incentive* as above will be used to optimize response rate.

**5.2.9 Outcomes**

Data for our outcomes will be collected from the **Ministry of Health mandatory 2-page Antenatal Records**, by chart abstraction using a Data Collection Form by the research staff a few weeks after the anticipated date of birth. Our **primary outcome will be** **total pregnancy weight gain**. [Justification: pregnancy weight gain was chosen as it is the outcome focused on clinically, directed by guidelines^5;6^. Weight is recorded on the Ministry of Health Antenatal Records at each antenatal visit (97% of visits^128^).] We will calculate ***total pregnancy weight gain*: we will subtract pre-pregnancy weight from the final measured weight** (both are available on the Antenatal Records^[[2]](#footnote-2)^). We will determine whether weight gain was “above” versus “within” the guidelines for BMI class, as per the guidelines ^5;6^.

Our **secondary outcomes** are: i) **1^st^ trimester weight gain** (at 13 weeks) **and 2^nd^ trimester weight gain** (at 28 weeks); ii), the **stability** **of determinants** in early versus late pregnancy (Questionnaire 1 versus 2); and iii) exploratory outcomes such as mode of delivery and birthweight.

**5.2.10 Plan for Data Analysis**

Data will be summarized using *descriptive statistics*: frequencies and proportions for categorical data and means/standard deviations or medians/interquartile ranges for normally and non-normally distributed continuous data, respectively. We will examine predictors of **total** **pregnancy weight gain, our primary outcome**, 1) 2 categorically (*above* versus *within* the guidelines) using **traditional logistic regression analysis to predict the risk of excess weight gain at an individual woman’s level**, as is done clinically and in the literature. We will begin by examining the relation of each potential determinant (initially continuously and then dichotomously as is typical in prenatal clinical care) with using *univariate logistic regression*. We will explore correlations between potential predictor variables using Pearson or Spearman correlation coefficients (for normally and not normally distributed variables, respectively). If there is a high correlation (correlation coefficient ≥ 0.70), the most psychologically or biologically relevant one will be chosen. We are interested in exploring complex interactions between predictors, hence we will test for *effect modification* (interaction effects) of a few psychologically or biologically meaningful variables, such as dietary restraint and impulsivity according to pre-pregnancy BMI. Potential predictor variables with a *p* <0.10 as well as interaction terms that are deemed psychologically or biologically meaningful will then be subjected to an automated *stepwise multivariable logistic regression* with criteria of p<0.05 for entry and removal, using a rule of 10 events per variable^132^.

We will assess the predictive ability of the resulting multivariable model using internal **validation**. Specifically, we will use 2/3 of our data to develop the model (*training data set*) and the remaining 1/3 to validate the model (*testing data set*). We will assess performance with measures including area under the receiver operating characteristic curve (AUC ROC) and the Hosmer-Lemeshow statistic.

**5.2.11 Our sample size calculation** is based on the following data:

1) We begin with the standard rule of “*10 events per variable* (EPV)” in the regression model^132^.

2) We estimate, based on our pilot study^29^, including *8 predictor variables* in the model.

3) However, as the non-pregnant weight literature recommends *stratification by excess vs normal weight*^38;80-82^, we will initially also include a potential stratification variable and an interaction term per predictor variable, yielding a total of (1+ 8*2=)17 predictor variables (sample size requires 17*10 EPV=170 women with the outcome of interest)

*Logit (p_i_)= β_0_ + β_stratification_Prepregnancy BMI category + β_1_ X_1_ + β_1 INTERACTION_ X_1*_Prepregnancy BMI category*

Takes into account potential stratification Takes into account potential interaction

*+ β_2_ X_2_ + β_2 INTERACTION_ X_2*_Prepregnancy BMI category + . . . . etc*

4) Given that 20%^3^-30%^2;4^ of women gain weight within the guidelines and 50%^1;2^-60%^3;4^ of women gain above the guidelines^29^, the smaller group is women gaining within. Guided by our pilot data^29^ in which *25% gained within and 50% gained above*, we would require 170 women gaining within and 340 gaining above (170+340=510 women gaining either within or above).

5) Since women who gain below the guidelines (25%) will not be included in the primary analysis, we need to recruit a larger sample size (hence the effective sample size until this point is 510/0.75=680).

6) *Missing data:* in our pilot, few predictors had >2% missing data and weight gain was available for 97.3%, hence, we have accordingly increased the sample size^138^ (680/0.97=694). We will make every effort to decrease missing data as described in Section 5.2.8, however, should there be missing data beyond this, to maintain feasibility and contain costs, we will perform *multiple imputation*.

7*) Model validation*: we will use the standard 2/3 to 1/3 ratio for training data to testing data.

Hence, we would require 694 participants for the training data set, and 348 for the testing data set, i.e.

a total of 1042 participants.

Reference List

(1) Park S, Sappenfield WM, Bish C, Salihu H, Goodman D, Bensyl DM. Assessment of the Institute of Medicine recommendations for weight gain during pregnancy: Florida, 2004-2007. *Matern Child Health J* 2011; 15(3):289-301.

(2) Kowal C, Kuk J, Tamim H. Characteristics of weight gain in pregnancy among Canadian women. *Matern Child Health J* 2012; 16(3):668-676.

(3) Durie DE, Thornburg LL, Glantz JC. Effect of second-trimester and third-trimester rate of gestational weight gain on maternal and neonatal outcomes. *Obstet Gynecol* 2011; 118(3):569-575.

(4) Simas TA, Waring ME, Liao X, Garrison A, Sullivan GM, Howard AE et al. Prepregnancy weight, gestational weight gain, and risk of growth affected neonates. *J Womens Health (Larchmt )* 2012; 21(4):410-417.

(5) IOM (Institute of Medicine). Weight Gain During Pregnancy: Reexamining the Guidelines. 2009. Washington, DC, The National Academies Press.

(6) Health Canada. Canadian Gestational Weight Gain Recommendations. Health Canada . 2009. 22-6-2010.

(7) Thorsdottir I, Torfadottir JE, Birgisdottir BE, Geirsson RT. Weight gain in women of normal weight before pregnancy: complications in pregnancy or delivery and birth outcome. *Obstet Gynecol* 2002; 99(5 Pt 1):799-806.

(8) Cedergren M. Effects of gestational weight gain and body mass index on obstetric outcome in Sweden. *Int J Gynaecol Obstet* 2006; 93(3):269-274.

(9) Rooney BL, Schauberger CW. Excess pregnancy weight gain and long-term obesity: one decade later. *Obstet Gynecol* 2002; 100(2):245-252.

(10) Johnston EM. Weight changes during pregnancy and the postpartum period. *Prog Food Nutr Sci* 1991; 15(3):117-157.

(11) Rooney BL, Schauberger CW, Mathiason MA. Impact of perinatal weight change on long-term obesity and obesity-related illnesses. *Obstet Gynecol* 2005; 106(6):1349-1356.

(12) Bergmann RL, Richter R, Bergmann KE, Plagemann A, Brauer M, Dudenhausen JW. Secular trends in neonatal macrosomia in Berlin: influences of potential determinants. *Paediatr Perinat Epidemiol* 2003; 17(3):244-249.

(13) Nohr EA, Vaeth M, Baker JL, Sorensen TI, Olsen J, Rasmussen KM. Combined associations of prepregnancy body mass index and gestational weight gain with the outcome of pregnancy. *Am J Clin Nutr* 2008; 87(6):1750-1759.

(14) Gillman MW, Rifas-Shiman S, Berkey CS, Field AE, Colditz GA. Maternal gestational diabetes, birth weight, and adolescent obesity. *Pediatrics* 2003; 111(3):e221-e226.

(15) Nehring I, Schmoll S, Beyerlein A, Hauner H, von KR. Gestational weight gain and long-term postpartum weight retention: a meta-analysis. *Am J Clin Nutr* 2011; 94(5):1225-1231.

(16) Mannan M, Doi SA, Mamun AA. Association between weight gain during pregnancy and postpartum weight retention and obesity: a bias-adjusted meta-analysis. *Nutr Rev* 2013; 71(6):343-352.

(17) Siega-Riz AM, Viswanathan M, Moos MK, Deierlein A, Mumford S, Knaack J et al. A systematic review of outcomes of maternal weight gain according to the Institute of Medicine recommendations: birthweight, fetal growth, and postpartum weight retention. *Am J Obstet Gynecol* 2009; 201(4):339.e1-339.e14.

(18) Schellong K, Schulz S, Harder T, Plagemann A. Birth weight and long-term overweight risk: systematic review and a meta-analysis including 643,902 persons from 66 studies and 26 countries globally. *PLoS One* 2012; 7(10):e47776.

(19) Yu ZB, Han SP, Zhu GZ, Zhu C, Wang XJ, Cao XG et al. Birth weight and subsequent risk of obesity: a systematic review and meta-analysis. *Obes Rev* 2011; 12(7):525-542.

(20) Guelinckx I, Devlieger R, Mullie P, Vansant G. Effect of lifestyle intervention on dietary habits, physical activity, and gestational weight gain in obese pregnant women: a randomized controlled trial. *American Journal of Clinical Nutrition* 2010; 91(2):373-380.

(21) Gray-Donald K, Robinson E, Collier A, David K, Renaud L, Rodrigues S. Intervening to reduce weight gain in pregnancy and gestational diabetes mellitus in Cree communities: an evaluation.[see comment]. *CMAJ Canadian Medical Association Journal 163(10):1247-51,* 2000.

(22) Kinnunen TI, Pasanen M, Aittasalo M, Fogelholm M, Hilakivi-Clarke L, Weiderpass E et al. Preventing excessive weight gain during pregnancy - a controlled trial in primary health care. *European Journal of Clinical Nutrition* 2007; 61(7):884-891.

(23) Dodd JM, Crowther CA, Robinson JS. Dietary and lifestyle interventions to limit weight gain during pregnancy for obese or overweight women: a systematic review. [Review] [31 refs]. *Acta Obstetricia et Gynecologica Scandinavica* 2008; 87(7):702-706.

(24) Gardner B, Wardle J, Poston L, Croker H. Changing diet and physical activity to reduce gestational weight gain: a meta-analysis. *Obes Rev* 2011; 12(7):e602-e620.

(25) Skouteris H, Hartley-Clark L, McCabe M, Milgrom J, Kent B, Herring SJ et al. Preventing excessive gestational weight gain: a systematic review of interventions. *Obes Rev* 2010; 11(11):757-768.

(26) Hill B, Skouteris H, McCabe M, Milgrom J, Kent B, Herring SJ et al. A conceptual model of psychosocial risk and protective factors for excessive gestational weight gain. *Midwifery* 2013; 29(2):110-114.

(27) Kapadia M, Gaston A, Van Blyderveen S, Schmidt L, Beyene J, McDonald H et al. Psychological antecedents of excessive pregnancy weight gain: a systematic review. *BMC Pregnancy & Childbirth* 2014; Response to reviews submitted February 3, 2015, MS: 2119845510147141.

(28) McDonald S, Park C, Timm V, Schmidt L, Neupane B, Beyene J. What psychological, physical, lifestyle and knowledge factors are associated with excess or inadequate weight gain during pregnancy? A cross-sectional survey. *J Obstet Gynaecol Can* 2013; 35(11):1071-1082.

(29) Park C, Krebs L, Lutsiv O, Van Blyderveen S, Schmidt L, Beyene J et al. Binge eating predicts excess gestational weight gain: a pilot prospective cohort study. *Journal of Obstetrics & Gyneoclogy of Canada* 2015; Accepted, January 9, 2015, Manuscript ID 14-12-240 .

(30) Ajzen I. From intentions to actions: A theory of planned behavior. In J.Kuhl &JBE, editor. 11-39. 1985. Berlin, Springer. Springer series in social psychology.

(31) Baranowski T, Cullen KW, Nicklas T, Thompson D, Baranowski J. Are current health behavioral change models helpful in guiding prevention of weight gain efforts? *Obes Res* 2003; 11 Suppl:23S-43S.

(32) Armitage CJ, Conner M. Efficacy of the Theory of Planned Behaviour: a meta-analytic review. *Br J Soc Psychol* 2001; 40(Pt 4):471-499.

(33) Conner M, Armitage CJ. Extending the Theory of Planned Behavior: A Review and Avenues for Further Research. *Journal of Applied Social Psychology* 1998; 28(15):1429-1464.

(34) Godin G, Kok G. The theory of planned behavior: a review of its applications to health-related behaviors. *Am J Health Promot* 1996; 11(2):87-98.

(35) Bray GA. Classification and Evaluation of the Overweight Patient. In: Bray GA, Bouchard C, editors. Handbook of Obesity: Clinical Applications. 2 ed. New York: Marcel Dekker, Inc.; 4 A.D. 1-32.

(36) Yankovski SZ, Stunkard AJ. Obesity and Eating Disorders. In: Bray GA, Bouchard C, editors. Handbook of Obesity: Clinical Applications. 2 ed. New York: Marcel Dekker Inc.; 2004. 201-225.

(37) Stunkard AJ. Binge-Eating Disorder and Night-Eating Syndrome. In: Wadden TA, Stunkard AJ, editors. Handbook of Obesity Treatment. New York: The Guilford Press; 2002. 107-124.

(38) Spitzer RL, Devlin M, Walsh BT, Hasin D, Wing R, Marcus M et al. Binge eating disorder: A multisite field trial of the diagnostic criteria. *Int J Eat Disord* 1992; 11(3):191-203.

(39) van Strien T, Frijters JER, Bergers GPA, Defares PB. The Dutch Eating Behavior Questionnaire (DEBQ) for assessment of restrained, emotional, and external eating behavior. *Int J Eat Disord* 1986; 5:295-315. doi: 10.1002/1098-108X(198602)5:2<295::AID-EAT2260050209>3.0.CO;2-T.

(40) Fell DB, Joseph KS, Dodds L, Allen AC, Jangaard K, Van den HM. Changes in maternal characteristics in Nova Scotia, Canada from 1988 to 2001. *Can J Public Health* 2005; 96(3):234-238.

(41) Gunderson EP, Abrams B. Epidemiology of gestational weight gain and body weight changes after pregnancy. *Epidemiol Rev* 2000; 22(2):261-274.

(42) Bradley PJ. Conditions recalled to have been associated with weight gain in adulthood. *Appetite* 1985; 6(3):235-241.

(43) Thorsdottir I, Torfadottir JE, Birgisdottir BE, Geirsson RT. Weight gain in women of normal weight before pregnancy: complications in pregnancy or delivery and birth outcome. *Obstet Gynecol* 2002; 99(5 Pt 1):799-806.

(44) Gilbert WM, Nesbitt TS, Danielsen B. Associated factors in 1611 cases of brachial plexus injury. *Obstet Gynecol* 1999; 93(4):536-540.

(45) Gottlieb AG, Galan HL. Shoulder dystocia: an update. *Obstet Gynecol Clin North Am* 2007; 34(3):501-31, xii.

(46) Nesbitt TS, Gilbert WM, Herrchen B. Shoulder dystocia and associated risk factors with macrosomic infants born in California. *Am J Obstet Gynecol* 1998; 179(2):476-480.

(47) Oral E, Cagdas A, Gezer A, Kaleli S, Aydinli K, Ocer F. Perinatal and maternal outcomes of fetal macrosomia. *Eur J Obstet Gynecol Reprod Biol* 2001; 99(2):167-171.

(48) Martin RJ, Hausman GJ, Hausman DB. Regulation of adipose cell development in utero. *Proc Soc Exp Biol Med* 1998; 219(3):200-210.

(49) Birmingham CL, Muller JL, Palepu A, Spinelli JJ, Anis AH. The cost of obesity in Canada. *CMAJ* 1999; 160(4):483-488.

(50) Tjepkema M. Measured Obesity: Adult obesity inCanada: Measured height and weight. Statistics Canada, editor. 82-620-MWE2005001. 2005.

(51) McGinnis JM, Foege WH. Actual causes of death in the United States. *JAMA* 1993; 270(18):2207-2212.

(52) Ezzati M, Lopez AD, Rodgers A, Vander HS, Murray CJ. Selected major risk factors and global and regional burden of disease. *Lancet* 2002; 360(9343):1347-1360.

(53) Jung RT. Obesity as a disease. [Review] [50 refs]. *British Medical Bulletin 53(2):307-21,* 1997.

(54) World Health Organization. Obesity: preventing and managing the global epidemic. technical report series no 894. 2000. Geneva, The Organization.

(55) Herring SJ, Nelson DB, Davey A, Klotz AA, Dibble LV, Oken E et al. Determinants of excessive gestational weight gain in urban, low-income women. *Womens Health Issues* 2012; 22(5):e439-e446.

(56) Daemers DO, Wijnen HA, van Limbeek EB, Bude LM, de Vries RG. Patterns of gestational weight gain in healthy, low-risk pregnant women without co-morbidities. *Midwifery* 2013; 29(5):535-541.

(57) Koh H, Ee TX, Malhotra R, Allen JC, Tan TC, Ostbye T. Predictors and adverse outcomes of inadequate or excessive gestational weight gain in an Asian population. *J Obstet Gynaecol Res* 2013; 39(5):905-913.

(58) Olson CM, Strawderman MS. Modifiable behavioral factors in a biopsychosocial model predict inadequate and excessive gestational weight gain. *J Am Diet Assoc* 2003; 103(1):48-54.

(59) Muktabhant B, Lumbiganon P, Ngamjarus C, Dowswell T. Interventions for preventing excessive weight gain during pregnancy. *Cochrane Database Syst Rev* 2012; 4:CD007145.

(60) Dodd JM, Grivell RM, Crowther CA, Robinson JS. Antenatal interventions for overweight or obese pregnant women: a systematic review of randomised trials. *BJOG* 2010; 117(11):1316-1326.

(61) Thangaratinam S, Rogozinska E, Jolly K, Glinkowski S, Roseboom T, Tomlinson JW et al. Effects of interventions in pregnancy on maternal weight and obstetric outcomes: meta-analysis of randomised evidence. *BMJ* 2012; 344:e2088.

(62) Choi J, Fukuoka Y, Lee JH. The effects of physical activity and physical activity plus diet interventions on body weight in overweight or obese women who are pregnant or in postpartum: a systematic review and meta-analysis of randomized controlled trials. *Prev Med* 2013; 56(6):351-364.

(63) Wolff S, Legarth J, Vangsgaard K, Toubro S, Astrup A. A randomized trial of the effects of dietary counseling on gestational weight gain and glucose metabolism in obese pregnant women. *International Journal of Obesity* 2008; 32(3):495-501.

(64) Claesson IM, Sydsjo G, Brynhildsen J, Cedergren M, Jeppsson A, Nystrom F et al. Weight gain restriction for obese pregnant women: a case-control intervention study. *BJOG* 2008; 115(1):44-50.

(65) Dodd JM, Turnbull D, McPhee AJ, Deussen AR, Grivell RM, Yelland LN et al. Antenatal lifestyle advice for women who are overweight or obese: LIMIT randomised trial. *BMJ* 2014; 348:g1285.

(66) Polley BA, Wing RR, Sims CJ. Randomized controlled trial to prevent excessive weight gain in pregnant women. *International Journal of Obesity & Related Metabolic Disorders: Journal of the International Association for the Study of Obesity* 2002; 26(11):1494-1502.

(67) Olson CM, Strawderman MS, Reed RG. Efficacy of an intervention to prevent excessive gestational weight gain. *Am J Obstet Gynecol* 2004; 191(2):530-536.

(68) Hickey CA, Cliver SP, Goldenberg RL, McNeal SF, Hoffman HJ. Relationship of psychosocial status to low prenatal weight gain among nonobese black and white women delivering at term. *Obstet Gynecol* 1995; 86(2):177-183.

(69) Jokela M, Hintsanen M, Hakulinen C, Batty GD, Nabi H, Singh-Manoux A et al. Association of personality with the development and persistence of obesity: a meta-analysis based on individual-participant data. *Obes Rev* 2013; 14(4):315-323.

(70) Wright C, Bilder D, DeBlasis T, Mogul M, Rubin D, Shea JA. Psychosocial factors associated with gestational weight gain in a low-income cohort. *J Health Care Poor Underserved* 2013; 24(1):332-343.

(71) Cogswell ME, Scanlon KS, Fein SB, Schieve LA. Medically advised, mother's personal target, and actual weight gain during pregnancy. *Obstet Gynecol* 1999; 94(4):616-622.

(72) Sangi-Haghpeykar H, Lam K, Raine SP. Gestational weight gain among Hispanic women. *Matern Child Health J* 2014; 18(1):153-160.

(73) Conway R, Reddy S, Davies J. Dietary restraint and weight gain during pregnancy. *Eur J Clin Nutr* 1999; 53(11):849-853.

(74) Gratz K, Roemer L. Multidimensional assessment of emotion regulation and dysregulation: Development, factor structure, and initial validation of the difficulties in emotion regulation scale. *J Psychopathol Behav Assess* 2004; 26(1):41-54.

(75) Gross JJ, John OP. Individual differences in two emotion regulation processes: implications for affect, relationships, and well-being. *J Pers Soc Psychol* 2003; 85(2):348-362.

(76) Wadden TA, Phelan S. Behavioural Assessment of the Obese Patient. In: Wadden TA, Stunkard AJ, editors. Handbook of Obesity Treatment. New York: The Guilford Press; 2002. 186-228.

(77) Birketvedt GS, Florholmen J, Sundsfjord J, Osterud B, Dinges D, Bilker W et al. Behavioral and neuroendocrine characteristics of the night-eating syndrome. *JAMA* 1999; 282(7):657-663.

(78) Stunkard AJ. Eating patterns and obesity. *Psychiatr Q* 1959; 33:284-295.

(79) Rand CS, Macgregor AM, Stunkard AJ. The night eating syndrome in the general population and among postoperative obesity surgery patients. *Int J Eat Disord* 1997; 22(1):65-69.

(80) Ro O, Reas DL, Rosenvinge J. The impact of age and BMI on Eating Disorder Examination Questionnaire (EDE-Q) scores in a community sample. *Eat Behav* 2012; 13(2):158-161.

(81) Desai MN, Miller WC, Staples B, Bravender T. Risk factors associated with overweight and obesity in college students. *J Am Coll Health* 2008; 57(1):109-114.

(82) Neumark-Sztainer D, WALL M, Story M, Standish AR. Dieting and unhealthy weight control behaviors during adolescence: associations with 10-year changes in body mass index. *J Adolesc Health* 2012; 50(1):80-86.

(83) Vocks S, Tuschen-Caffier B, Pietrowsky R, Rustenbach SJ, Kersting A, Herpertz S. Meta-analysis of the effectiveness of psychological and pharmacological treatments for binge eating disorder. *Int J Eat Disord* 2010; 43(3):205-217.

(84) Whiteside U, Chen E, Neighbors C, Hunter D, Lo T, Larimer M. Difficulties regulating emotions: Do binge eaters have fewer strategies to modulate and tolerate negative affect? *Eat Behav* 2007; 8(2):162-169.

(85) Stice E. Risk and maintenance factors for eating pathology: a meta-analytic review. *Psychol Bull* 2002; 128(5):825-848.

(86) Isnard P, Michel G, Frelut ML, Vila G, Falissard B, Naja W et al. Binge eating and psychopathology in severely obese adolescents. *Int J Eat Disord* 2003; 34(2):235-243.

(87) Herpertz-Dahlmann B. Adolescent eating disorders: definitions, symptomatology, epidemiology and comorbidity. *Child Adolesc Psychiatr Clin N Am* 2009; 18(1):31-47.

(88) Dansky BS, Brewerton TD, Kilpatrick DG, O'Neil PM. The National Women's Study: relationship of victimization and posttraumatic stress disorder to bulimia nervosa. *Int J Eat Disord* 1997; 21(3):213-228.

(89) Brewerton TD, Stellefson EJ, Hibbs N, Hodges EL, Cochrane CE. Comparison of eating disorder patients with and without compulsive exercising. *Int J Eat Disord* 1995; 17(4):413-416.

(90) Pani PP, Maremmani I, Trogu E, Gessa GL, Ruiz P, Akiskal HS. Delineating the psychic structure of substance abuse and addictions: should anxiety, mood and impulse-control dysregulation be included? *J Affect Disord* 2010; 122(3):185-197.

(91) Ting WH, Huang CY, Tu YK, Chien KL. Association between weight status and depressive symptoms in adolescents: role of weight perception, weight concern, and dietary restraint. *Eur J Pediatr* 2012; 171(8):1247-1255.

(92) Krebs P, Prochaska JO, Rossi JS. A meta-analysis of computer-tailored interventions for health behavior change. *Prev Med* 2010; 51(3-4):214-221.

(93) Kroeze W, Werkman A, Brug J. A systematic review of randomized trials on the effectiveness of computer-tailored education on physical activity and dietary behaviors. *Ann Behav Med* 2006; 31(3):205-223.

(94) Broekhuizen K, Kroeze W, van Poppel MN, Oenema A, Brug J. A systematic review of randomized controlled trials on the effectiveness of computer-tailored physical activity and dietary behavior promotion programs: an update. *Ann Behav Med* 2012; 44(2):259-286.

(95) Hinton PS, Olson CM. Postpartum exercise and food intake: the importance of behavior-specific self-efficacy. *J Am Diet Assoc* 2001; 101(12):1430-1437.

(96) Wallston BS, Wallston KA, Kaplan GD, Maides SA. Development and validation of the health locus of control (HLC) scale. *J Consult Clin Psychol* 1976; 44(4):580-585.

(97) Chambliss CA, Murray EJ. Efficacy attribution, locus of control, and weight loss. *Cognitive Therapy and Research* 1979; 3(4):349-353.

(98) Noar SM, Benac CN, Harris MS. Does tailoring matter? Meta-analytic review of tailored print health behavior change interventions. *Psychol Bull* 2007; 133(4):673-693.

(99) Sohl SJ, Moyer A. Tailored interventions to promote mammography screening: a meta-analytic review. *Prev Med* 2007; 45(4):252-261.

(100) Civljak M, Stead LF, Hartmann-Boyce J, Sheikh A, Car J. Internet-based interventions for smoking cessation. *Cochrane Database Syst Rev* 2013; 7:CD007078.

(101) Statistics Canada. Births and total fertility rate, by province and territory. Accessed at <http://www.statcan.gc.ca/tables-tableaux/sum-som/l01/cst01/hlth85a-eng.htm> on July 2, 2013. 2013.

(102) The Government of Ontario. Ontario Regional Boundaries. Accessed at <http://www.gojobs.gov.on.ca/regions.asp> on July 19, 2013. 2013.

(103) Public Health Agency of Canada. What Mothers Say: The Canadian Maternity Experiences Survey. Ottawa, 2009. Available at: <http://www.phac-aspc.gc.ca/rhs-ssg/pdf/survey-eng.pdf>. 2009.

(104) Tong S, Kaur A, Walker SP, Bryant V, Onwude JL, Permezel M. Miscarriage risk for asymptomatic women after a normal first-trimester prenatal visit. *Obstet Gynecol* 2008; 111(3):710-714.

(105) Makrydimas G, Sebire NJ, Lolis D, Vlassis N, Nicolaides KH. Fetal loss following ultrasound diagnosis of a live fetus at 6-10 weeks of gestation. *Ultrasound Obstet Gynecol* 2003; 22(4):368-372.

(106) Boyd PA, Tondi F, Hicks NR, Chamberlain PF. Autopsy after termination of pregnancy for fetal anomaly: retrospective cohort study. *BMJ* 2004; 328(7432):137.

(107) Maggard MA, Yermilov I, Li Z, Maglione M, Newberry S, Suttorp M et al. Pregnancy and fertility following bariatric surgery: a systematic review. *JAMA* 2008; 300(19):2286-2296.

(108) Swann RA, Von HA, Torgersen L, Gendall K, Reichborn-Kjennerud T, Bulik CM. Attitudes toward weight gain during pregnancy: results from the Norwegian mother and child cohort study (MoBa). *Int J Eat Disord* 2009; 42(5):394-401.

(109) Ekeus C, Lindberg L, Lindblad F, Hjern A. Birth outcomes and pregnancy complications in women with a history of anorexia nervosa. *BJOG* 2006; 113(8):925-929.

(110) Kendall A, Olson CM, Frongillo EA, Jr. Evaluation of psychosocial measures for understanding weight-related behaviors in pregnant women. *Ann Behav Med* 2001; 23(1):50-58.

(111) Williamson DA, Womble LG, Zucker NL, Reas DL, White MA, Blouin DC et al. Body image assessment for obesity (BIA-O): development of a new procedure. *Int J Obes Relat Metab Disord* 2000; 24(10):1326-1332.

(112) Fowles ER, Feucht J. Testing the barriers to healthy eating scale. *West J Nurs Res* 2004; 26(4):429-443.

(113) Parmenter K, Wardle J. Development of a general nutrition knowledge questionnaire for adults. *Eur J Clin Nutr* 1999; 53(4):298-308.

(114) Rini CK, Dunkel-Schetter C, Wadhwa PD, Sandman CA. Psychological adaptation and birth outcomes: the role of personal resources, stress, and sociocultural context in pregnancy. *Health Psychol* 1999; 18(4):333-345.

(115) Garner D, Olmstead M, Polivy J. Development and validation of a multidimensional eating disorder inventory for anorexia nervosa and bulimia. *Int J Eat Disord* 1983; 2(2):15-34.

(116) Dishman RK, Ickes W. Self-motivation and adherence to therapeutic exercise. *J Behav Med* 1981; 4(4):421-438.

(117) Gosling SD, Rentfrow PJ, Swann WB Jr. A very brief measure of the Big-Five personality domains. *Journal of Research in Personality* 2003; 37:504-528.

(118) Herman C, Polivy J. Restrained eating. in A. Stunkard (Ed.), Obesity. Phildelphia, PA: Saunders; 1980. 208-225.

(119) Block G, Hartman AM, Naughton D. A reduced dietary questionnaire: development and validation. *Epidemiology* 1990; 1(1):58-64.

(120) Schmidt MD, Freedson PS, Pekow P, Roberts D, Sternfeld B, Chasan-Taber L. Validation of the Kaiser Physical Activity Survey in pregnant women. *Med Sci Sports Exerc* 2006; 38(1):42-50.

(121) Ainsworth BE, Sternfeld B, Richardson MT, Jackson K. Evaluation of the kaiser physical activity survey in women. *Med Sci Sports Exerc* 2000; 32(7):1327-1338.

(122) Overduin J, Jansen A. A new scale for use in non-clinical research into disinhibitive eating. *Pers Individ Dif* 1996; 20(6):669-677.

(123) Crane JM, White J, Murphy P, Burrage L, Hutchens D. The effect of gestational weight gain by body mass index on maternal and neonatal outcomes. *J Obstet Gynaecol Can* 2009; 31(1):28-35.

(124) Norman G, Streiner D. PDQ Statistics (3rd ed.). Toronto: B.C. Decker; 2003.

(125) Dillman Don A, Smyth Jolene D, Christian Leah M. Implementation Procedures. Internet, Mail, and Mixed-Mode Surveys. The Tailored Design Method. 3rd ed. Hoboken, New Jersey, USA: John Wiley & Sons; 2009. 234-299.

(126) Kingston D, Sword W, Krueger P, Hanna S, Markle-Reid M. Life Course Pathways to Prenatal Maternal Stress. *J Obstet Gynecol Neonatal Nurs 2012 Jun 27* 2012; 41: 609-626. doi: 10.1111/j.1552-6909.2012.01381.x .

(127) Ziegenfuss JY, Burmeister KR, Harris A, Holubar SD, Beebe TJ. Telephone follow-up to a mail survey: when to offer an interview compared to a reminder call. *BMC Med Res Methodol* 2012; 12:32.

(128) McDonald SD, Machold CA, Marshall L, Kingston D. Documentation of guideline adherence in antenatal records across maternal weight categories. *BMC Pregnancy and Childbirth, Under Review, November 28, 2013 Manuscript number MS: 1896943037114260* 2013.

(129) Schieve LA, Perry GS., Cogswell ME, Scanion KS, Rosenberg D, Carmichael S et al. Validity of self-reported pregnancy delivery weight: an analysis of the 1988 National Maternal and Infant Health Survey. NMIHS Collaborative Working Group. *American Journal of Epidemiology 150(9):947-56,* 1999.

(130) Lederman SA, Paxton A. Maternal reporting of prepregnancy weight and birth outcome: consistency and completeness compared with the clinical record. *Matern Child Health J* 1998; 2(2):123-126.

(131) Johnston EM. Weight changes during pregnancy and the postpartum period. *Prog Food Nutr Sci* 1991; 15(3):117-157.

(132) Peduzzi P, Concato J, Kemper E, Holford TR, Feinstein AR. A simulation study of the number of events per variable in logistic regression analysis. *J Clin Epidemiol* 1996; 49(12):1373-1379.

(133) G.W.Milligan. Clustering validation: results and implications for applied analyses, in: Arabie P, Hubert LJ and De Soete G (eds), Clustering and Classifcation. *World Scientific, Singapore* 1996;341-375.

(134) Tritchler D, Parkhomenko E, Beyene J. Filtering genes for cluster and network analysis. *BMC Bioinformatics* 2009; 10:193.

(135) Tritchler D, Fallah S, Beyene J. A spectral clustering method for microarray data. *Computational Satatistics and Data Analysis* 2005; 49:63-76.

(136) Wen LM, Simpson JM, Rissel C, Baur LA. Maternal "junk food" diet during pregnancy as a predictor of high birthweight: findings from the healthy beginnings trial. *Birth* 2013; 40(1):46-51.

(137) Olafsdottir AS, Skuladottir GV, Thorsdottir I, Hauksson A, Steingrimsdottir L. Maternal diet in early and late pregnancy in relation to weight gain. *International Journal of Obesity 30(3):492-9, 2006 Mar* 2006;(3):492-499.

(138) Kelsey J, Whittemore A, Evans A, Thompson W. Methods in Observational Epidemiology (2nd ed.). Cary, North Carolina: Oxford University Press; 1996.

(139) Guadagnoli E, Velicer WF. Relation of sample size to the stability of component patterns. *Psychol Bull* 1988; 103(2):265-275.

(140) Osborne JW, Costello AB. Sample size and subject to item ratio in principal components analysis. *Practical Assessment, Research & Evaluation* 2004; Retrieved July 31, 2013 from <http://PAREonline.net/getvn.aasp?v=9&n=11>.

(141) McDonald SD, Pullenayegum E, Taylor V, Lutsiv O, Bracken K, Good C et al. Despite 2009 guidelines few women report being counseled correctly about weight gain during pregnancy. *Am J Obstet Gynecol* 2011; 205(4):333.e1-6 (Editor's Choice).

(142) Lutsiv O, Bracken K, Pullenayegum E, Sword W, Taylor VH, McDonald SD. Little congruence between health care provider and patient perceptions of counselling on gestational weight gain. *J Obstet Gynaecol Can* 2012; 34(6):518-524.

(143) McDonald SD, Pullenayegum E, Bracken K, Chen AM, McDonald H, Malott A et al. Comparison of midwifery, family medicine and obstetric patients' understanding of weight gain during pregnancy: a minority of women report correct counseling . *J Obstet Gynaecol Can* 2012; 34(2):129-135.

(144) Sword W, Watt S, Krueger P, Thabane L, Landy CK, Farine D et al. The Ontario Mother and Infant Study (TOMIS) III: a multi-site cohort study of the impact of delivery method on health, service use, and costs of care in the first postpartum year. *BMC Pregnancy Childbirth* 2009; 9:16.

(145) Sword W, Heaman MI, Brooks S, Tough S, Janssen PA, Young D et al. Women's and care providers' perspectives of quality prenatal care: a qualitative descriptive study. *BMC Pregnancy Childbirth* 2012; 12:29.

(146) Morais M, Mehta C, Murphy K, Shah PS, Giglia L, Smith PA et al. How Often are Late Preterm Births Due to Non-Evidence Based Practices?: Analysis from Two tertiary referral Centres in a Nationalized Health Care System--A Retrospective Cohort Study. *BJOG* 2013; 120(12):1508-1514 doi: 10.1111/1471-0528.12401.

(147) Thorne S. Interpretive Description. Walnut Creek, CA: Left Coast Press Inc; 2008.

(148) Jack SM, Dobbins M, Sword W, Novotna G, Brooks S, Lipman EL et al. Evidence-informed decision-making by professionals working in addiction agencies serving women: a descriptive qualitative study. *Subst Abuse Treat Prev Policy* 2011; 6:29.

(149) Sword W, Niccols A, Dobbins M, Henderson J, Brooks S. Participant perspectives of a knowledge broker intervention for addiction agencies serving women in Canada. *Manuscript in preparation* 2014.

(150) Sandelowski M. Sample size in qualitative research. *Res Nurs Health* 1995; 18(2):179-183.

(151) Ritchie J, Lewis J, Elam G. Qualitative Research Practice: A Guide for Social Science Students and Researchers. In: Ritchie J, Lewis J, editors. London: Sage Publications; 2003.

(152) Patton MQ. Qualitative Research & Evaluation Methods (3rd Edition). 3rd ed. Thousand Oaks: Sage Publications; 2002.

(153) Sturges J, Hanrahan K. Comparing telephone and face-to-face qualitative interviewing: A research note. *Qual Res* 2004; 4(1):107-118.

(154) Novick G. Is there a bias against telephone interviews in qualitative research? *Res Nurs Health* 2008; 31(4):391-398.

(155) Strauss A, Corbin J. Basics of qualitative research. 2nd ed. Thousand Oaks, CA: Sage Publications; 1998.

(156) Sword W, Clark AM, Hegadoren K, Brooks S, Kingston D. The complexity of postpartum mental health and illness: a critical realist study. *Nurs Inq* 2012; 19(1):51-62.

(157) Sword W, Busser D, Ganann R, McMillan T, Swinton M. Women's care-seeking experiences after referral for postpartum depression. *Qual Health Res* 2008; 18(9):1161-1173.

(158) Vanstone M, Yacoub K, Giacomini M, Hulan D, McDonald S. Women's experiences of publicly-funded Non-Invasive prenatal Testing in Ontario, Canada: Considerations for health technology policy-making. *Social Science & Medicine* 2015; Submitted, January 28, 2015. Manuscript ID: SSM-D-15-00257.

(159) Kapadia MZ, Gaston A, Van Blyderveen S, Schmidt L, Beyene J, McDonald H et al. Psychological factors and trimester-specific gestational weight gain: a systematic review. *J Psychosom Obstet Gynaecol* 2014;1-8.

(160) Han Z, Mulla S, Beyene J, Liao G, McDonald SD, Knowledge Synthesis Group. Maternal Underweight and the risk of preterm birth and low birth weight: A systematic review and meta-analyses. *Int J of Epidemiology* 2011; 40(1):65-101.

(161) McDonald SD, Han Z, Mulla S, Beyene J. Maternal overweight and obesity and the risk of preterm birth and low birth weight: a systematic review and meta-analyses. *BMJ* 2010; BMJ.2010 Jul 20;341:c3428. doi: 10.1136/bmj.c3428.

(162) McDonald SD, Han Z, Mulla S, Lutsiv O, Lee T, Beyene J et al. High gestational weight gain and the risk of preterm birth and low birth weight: a systematic review and meta-analyses. *J Obstet Gynaecol Can* 2011; 33(12):1223-1233.

(163) Han Z, Lutsiv O, Mulla S, Rosen A, Beyene J, McDonald S et al. Low gestational weight gain and the risk of preterm birth and low birthweight: a systematic review and meta-analyses. *Acta Obstet Gynecol Scand 2011 Sept90(9)935-54* 2011.

(164) Sword W, Niccols A, Yousefi-Nooraie R, Dobbins M, Lipman E, Smith P. Partnerships Among Canadian Agencies Serving Women with Substance Abuse Issues and Their Children. *Int J Ment Health Addict* 2013; 11(3):344-357.

(165) Kingston D, Dennis CL, Sword W. Exploring breast-feeding self-efficacy. *J Perinat Neonatal Nurs* 2007; 21(3):207-215.

(166) Sword W, Watt S, Krueger P. Postpartum health, service needs, and access to care experiences of immigrant and Canadian-born women. *J Obstet Gynecol Neonatal Nurs* 2006; 35(6):717-727.

(167) Sword WA, Krueger PD, Watt MS. Predictors of acceptance of a postpartum public health nurse home visit: findings from an Ontario survey. *Can J Public Health* 2006; 97(3):191-196.

(168) Sword W, Watt S. Learning needs of postpartum women: does socioeconomic status matter? *Birth* 2005; 32(2):86-92.

(169) Peterson WE, Charles C, DiCenso A, Sword W. The Newcastle Satisfaction with Nursing Scales: a valid measure of maternal satisfaction with inpatient postpartum nursing care. *J Adv Nurs* 2005; 52(6):672-681.

(170) Sword W, Niccols A, Fan A. "New Choices" for women with addictions: perceptions of program participants. *BMC Public Health* 2004; 4:10.

(171) Vanstone M, Kinsella EA. Critical reflection and prenatal screening public education materials: a metaphoric textual analysis. *Reflective Practice* 2010; 11(4):451-467.

(172) Vanstone M, Giacomini M, Smith A, Brundisini F, DeJean D, Winsor S. How diet modification challenges are magnified in vulnerable or marginalized people with diabetes and heart disease: a systematic review and qualitative meta-synthesis. *Ont Health Technol Assess Ser* 2013; 13(14):1-40.

(173) Couturier J, Kimber M, Jack S, Niccols A, Van Blyderveen S, McVey G. Understanding the uptake of family-based treatment for adolescents with anorexia nervosa: therapist perspectives. *Int J Eat Disord* 2013; 46(2):177-188.

(174) Couturier J, Van Blyderveen S. Challenges in the Assessment and Diagnosis of Eating Disorders in Childhood and Adolescence given Current Diagnostic and Assessment Instruments. The Oxford Handbook of Developmental Perspectives on Child and Adolescent Eating Disorders (Ed. James Lock). Oxford, England: 2011.

(175) Van Lieshout RJ, Schmidt LA, Robinson M, Niccols A, Boyle MH. Maternal pre-pregnancy body mass index and offspring temperament and behavior at 1 and 2 years of age. *Child Psychiatry Hum Dev* 2013; 44(3):382-390.

(176) Leung E, Tasker SL, Atkinson L, Vaillancourt T, Schulkin J, Schmidt LA. Perceived maternal stress during pregnancy and its relation to infant stress reactivity at 2 days and 10 months of postnatal life. *Clin Pediatr (Phila)* 2010; 49(2):158-165.

(177) Mathewson KJ, Schmidt LA, Miskovic V, Santesso DL, Duku E, McCabe RE et al. Does respiratory sinus arrhythmia (RSA) predict anxiety reduction during cognitive behavioral therapy (CBT) for social anxiety disorder (SAD)? *Int J Psychophysiol* 2013; 88(2):171-181.

(178) Boyle MH, Miskovic V, Van LR, Duncan L, Schmidt LA, Hoult L et al. Psychopathology in young adults born at extremely low birth weight. *Psychol Med* 2011; 41(8):1763-1774.

(179) Edison SC, Evans MA, McHolm AE, Cunningham CE, Nowakowski ME, Boyle M et al. An investigation of control among parents of selectively mute, anxious, and non-anxious children. *Child Psychiatry Hum Dev* 2011; 42(3):270-290.

(180) Miskovic V, Ashbaugh AR, Santesso DL, McCabe RE, Antony MM, Schmidt LA. Frontal brain oscillations and social anxiety: a cross-frequency spectral analysis during baseline and speech anticipation. *Biol Psychol* 2010; 83(2):125-132.

(181) Schmidt LA, Miskovic V, Boyle M, Saigal S. Frontal electroencephalogram asymmetry, salivary cortisol, and internalizing behavior problems in young adults who were born at extremely low birth weight. *Child Dev* 2010; 81(1):183-199.

(182) Cairney J, Wade TJ. Correlates of body weight in the 1994 National Population Health Survey. *Int J Obes Relat Metab Disord* 1998; 22(6):584-591.

(183) Cairney J, Ostbye T. Time since immigration and excess body weight. *Can J Public Health* 1999; 90(2):120-124.

(184) Cairney J, Kwan M, Veldhuizen S, Faulkner GE. Who Uses Exercise as a Coping Strategy for Stress? Results From a National Survey of Canadians. *J Phys Act Health* 2013.

(185) Granek L, Rosenberg-Yunger ZR, Dix D, Klaassen RJ, Sung L, Cairney J et al. Caregiving, single parents and cumulative stresses when caring for a child with cancer. *Child Care Health Dev* 2012.

(186) Rueda S, Raboud J, Rourke SB, Bekele T, Bayoumi A, Lavis J et al. Influence of employment and job security on physical and mental health in adults living with HIV: cross-sectional analysis. *Open Med* 2012; 6(4):e118-e126.

(187) Mackenzie CS, Reynolds K, Cairney J, Streiner DL, Sareen J. Disorder-specific mental health service use for mood and anxiety disorders: associations with age, sex, and psychiatric comorbidity. *Depress Anxiety* 2012; 29(3):234-242.

(188) Ross LE, Villegas L, Dennis CL, Bourgeault IL, Cairney J, Grigoriadis S et al. Rural residence and risk for perinatal depression: a Canadian pilot study. *Arch Womens Ment Health* 2011; 14(3):175-185.

(189) Wade TJ, Veldhuizen S, Cairney J. Prevalence of psychiatric disorder in lone fathers and mothers: examining the intersection of gender and family structure on mental health. *Can J Psychiatry* 2011; 56(9):567-573.

(190) Hajna S, Liu J, LeBlanc PJ, Faught BE, Merchant AT, Cairney J et al. Association between body composition and conformity to the recommendations of Canada's Food Guide and the Dietary Approaches to Stop Hypertension (DASH) diet in peri-adolescence. *Public Health Nutr* 2012; 15(10):1890-1896.

(191) Beyene J, Atenafu EG, Hamid JS, To T, Sung L. Determining relative importance of variables in developing and validating predictive models. *BMC Med Res Methodol* 2009; 9:64.

(192) Statistics Canada. Births, estimates, by province and territory. Accessed at <http://www.statcan.gc.ca/tables-tableaux/sum-som/l01/cst01/demo04a-eng.htm> on February 18, 2014. 2013.

(193) Atkinson RL, Pietrobelli A, Uauy R, Macdonald IA. Are we attacking the wrong targets in the fight against obesity?: the importance of intervention in women of childbearing age. *Int J Obes (Lond)* 2012; 36(10):1259-1260.

1. Night eating, a diurnal response to stress with 25-50% of caloric intake after supper^77^, *increases in frequency with increasing weight*, occurring in 9% of people at an obesity clinic^78^ and 27% of people seeking obesity surgery^79^. [↑](#footnote-ref-1)
2. Maternal pre-pregnancy height & weight are self-reported in the clinical setting, with high correlation with measured values in pregnancy^129-131^. [↑](#footnote-ref-2)
