# Supplementary material for: Prediction of excess pregnancy weight gain using psychological, physical, and social predictors: A validated model in a prospective cohort study
Source: PLoS One. 2020 Jun 2;15(6):e0233774. doi: 10.1371/journal.pone.0233774 (PMC7266315; doi:10.1371/journal.pone.0233774)
Supplement: Attachment — Submitted filename: Response to Reviewers 2020Apr24 SUB.docx [file pone.0233774.s010.docx]

Dear Dr. Joerg Heber,

We appreciate the comments from the reviewers and have responded to each one below.

Review Comments to the Author

Please use the space provided to explain your answers to the questions above. You may also include additional comments for the author, including concerns about dual publication, research ethics, or publication ethics. (Please upload your review as an attachment if it exceeds 20,000 characters)

Reviewer #1: I’d like to thank the authors for answering the comments and incorporating the suggestions to the text whenever possible.
I strongly recommend that better prediction models are used in the future, but I agree with the answers provided to the comments on these models. I still have some considerations, though.

a. I strongly recommend that the sample is restricted to adults (Age >19 yo). The use of IOM recommendations and WHO cutoffs for pregnant adolescent women is debatable, so removing adolescents from the sample should be considered.

**Response:**

Thank you for this comment. As suggested, we performed the sensitivity analyses restricting the study sample to women aged 20 years and older. We found that this model has five predictors in common with the one derived from the whole sample. Although these models have some predictors in common and other predictors differ, they have comparable predictive powers.

In addition, the Institute of Medicine (IOM) weight gain guidelines (1) and the ACOG committee opinion, (2) and the Health Canada (3) suggest that the recommendations can be applied to both adults and adolescents.

Further, the IOM, on page 3-4, states "Evidence available since the IOM (1990) report is also insufficient to continue to support a modification of the GWG guidelines for adolescents (< 20 years old) during pregnancy. The committee also determined that prepregnancy BMI could be adequately categorized in adolescents by using the WHO cutoff points for adults, in part because of the impracticality of using pediatric growth charts in obstetric practices."

On page 251, it states “As discussed in Chapter 4, the committee was unable to identify sufficient evidence to continue to support a modification of the GWG guidelines for adolescents (females <20 years old) (Vishwanathan et al., 2008) (see Chapter 4)."

1. Institute of Medicine (US) and National Research Council (US) Committee to Reexamine IOM Pregnancy Weight Guidelines. Weight gain during pregnancy: reexamining the guidelines. Rasmussen KM, Yaktine AL, editors. Washington (DC): National Academies --Press (US); 2009. (The National Academies Collection: Reports funded by National Institutes of Health).

2. American College of Obstetricians and Gynecologists. ACOG Committee opinion no. 548: weight gain during pregnancy. Obstet Gynecol. 2013 Jan;121(1):210–2.

3. Canada H. Prenatal Nutrition Guidelines for Health Professionals: Gestational Weight Gain [Internet]. aem. 2010 [cited 2020 Apr 22]. Available from: https://www.canada.ca/en/health-canada/services/can

We, therefore, reported the results of the whole sample. We added a supplementary file reporting these data (S6 File). In statistical analyses section, we added the following statement:

***We performed sensitivity analyses by restricting the study sample to women who were aged 20 years and older and separately to women who gained weight within and above the recommendations.***

At the end of the Results, we added the following statement to explain the reasons to use the whole sample but not the restricted samples:

***In the sensitivity analyses, we found some selected predictors in common, although some differed, and these analyses yielded comparable predictive powers as that derived from the whole sample (S6 File). We focused on the analysis derived from the whole sample because: 1) in early pregnancy, we do not yet have the information to determine if a woman will gain weight above, within, or below the recommendations; and 2) the IOM guidelines recommend that both adults and adolescents should gain weight within the recommendations.***

b. I reinforce the need to compare excessive gestational weight gain using as reference the ‘adequate’ category, not the combination of insufficient + adequate. This would be a sensitive analysis only, to enrich the results and the sample size would not be substantially reduced since only 15% of women presented weight gain below the guidelines.

**Response:**

We performed the sensitivity analyses requested and found that excluding women who gained weight below the recommendations yielded a model with the predictive power comparable with the one derived from the whole sample. Although some predictors differed, there were some selected predictors in common among the models derived from the whole sample and the sample without inadequate weight gain. We planned to focus on prediction of excess GWG in early pregnancy, and in early pregnancy there is no way to know whether women will gain above, within, or below the recommendations. We, therefore, reported the results of the whole sample. We added a supplementary file reporting these data (S6 File). In the statistical analyses and results, we added the statements to explain the rationales why we report the results of the whole sample, as shown in our responses to the first comments.

c. If the 2-kg subtraction for first trimester weight was based on the results from the data, this should be added to the text. The IOM recommendations for first trimester vary between 0.5 – 2kg, so any amount in this interval could have been used. The justification for 2-kg was according to the pattern observed in a smaller sample of women and should be informed in the text, as it was in the answer to my comments.

**Response:**

We apologize for not adding the first-trimester weight gain data in the manuscript and added the following statement into the result section.

***The mean first trimester weight gain measured between 12-13 weeks of gestation was 1.83 ± 3.3 kg.***

d. The Introduction section is still focusing on psychological factors, not mentioning and exploring the other factors that might be associated with weight gain and were included in the models.

**Response:**

We apologize for this and have added some statements regarding planned weight gain, parity, and prepregnancy BMI in the introduction, as below:

***In addition to psychological factors, our pilot study found that planning to gain weight above the recommendations was associated with an increased likelihood of developing excess pregnancy weight gain compared with planning to gain weight within the recommendations.[27] Other studies also reported that some physical factors, including parity [10] and prepregnancy BMI,[28] might play a role in excess pregnancy weight gain.***

e. The fact that the AUC for the training set was low (< 0.70) is a problem the authors do not explained well enough. This low AUC may suggest that the prediction model is not good enough and its generalizability may be compromised. What are the solutions for that? What is the possible explanation?

**Response:**

We agree with the reviewer that the AUCs of our prediction model were moderate, i.e., 0.76 for training and 0.62 for testing datasets, respectively. We added some statements in limitations regarding the improvement of the predictive power in future studies.

***Future investigation is needed to assess whether the addition of some genetic or biochemical markers may improve the predictive power compared with the questionnaire-based model.***

Reviewer #2: All comments have been addressed, thus I am fully satisfied with the authors' response. The authors provided all data underlying the findings described in their manuscript.

**Response:**

We thank the reviewers for reviewing our manuscript and appreciate these comments.
